# Demyelination-induced glutamatergic imbalance mediates hippocampal Hyperexcitability

**DOI:** 10.1016/j.nbd.2025.107125

**Published:** 2025-09-24

**Authors:** Alyssa M. Anderson, Moyinoluwa Ajayi, Carrie R. Jonak, Shane Desfor, Joselyn Soto, Adrian Akhuetie, Devang Deshpande, Andrew Lapato, Devin K. Binder, Seema K. Tiwari-Woodruff

**Affiliations:** a Division of Biomedical Sciences, School of Medicine, University of California Riverside, Riverside, CA 92521, USA; b Center for Glial-Neuronal Interactions, School of Medicine, University of California Riverside, Riverside, CA 92521, USA

**Keywords:** Multiple sclerosis, Cuprizone diet, Glutamate, GABA, Seizures, Excitatory, Inhibitory, Demyelination, Electroencephalogram, NanoString RNA, Hippocampus

## Abstract

Chronic demyelination is a hallmark of multiple sclerosis (MS) and is associated with increased seizure susceptibility. In this study, we used the cuprizone (CPZ) diet induced demyelination model to investigate the progression of hippocampal demyelination and its impact on seizure activity and neurotransmitter dysregulation. Using EEG recordings, immunohistochemistry, Western blotting, ELISA, Golgi staining, and NanoString transcriptomics, we found progressive hippocampal demyelination accompanied by a striking increase in seizure incidence, from 38 % at 6 weeks to 88 % by 12 weeks. Structural degeneration of the CA1 pyramidal layer was marked by reduced dendritic arborization and loss of parvalbumin interneurons. Hippocampal glutamate levels increased as early as 3 weeks and remained elevated, with values (~2.2 μM) reaching excitotoxic thresholds, along with astrocyte reactivity (glial fibrillary acidic protein) and downregulation of astrocytic glutamate transporter-1, and glutamate aspartate Transporter-1 and modification of aquaporin-4 in CA1. Stratum pyramidal and stratum radiatum region-specific alterations in glutamate transporters and related enzymes (glutamine synthetase, glutamic acid decarboxylase 67, vesicular glutamate transporter 1), further supported neurotransmitter imbalance. Transcriptomic profiling revealed widespread downregulation of myelin, neuronal, astrocytic, glutamatergic, and GABAergic genes at 6 weeks, with partial recovery by 12 weeks. Together, these findings establish a mechanistic link between chronic hippocampal demyelination, glutamate dysregulation, and epileptogenesis offering potential molecular targets for therapeutic intervention in MS-associated epilepsy.

## 1. Introduction

MS is an autoimmune demyelinating and neurodegenerative disease of the central nervous system (CNS) (Walton et al., 2020; Attfield et al., 2022). People living with MS may experience a range of symptoms, including motor dysfunction, optic neuritis, cognitive decline, and seizures (Compston and Coles, 2002; Jakimovski et al., 2024). MS patients are three to six times more likely to experience seizures and face an elevated risk of developing epilepsy compared to the general population (Chou et al., 2019; Mirmosayyeb et al., 2021). Seizures may occur before the onset of MS in some individuals, while others develop them during disease progression (Catenoix et al., 2011; Calabrese et al., 2012; Nicholas et al., 2016; Mirmosayyeb et al., 2021). Magnetic resonance imaging (MRI) studies have shown that MS patients with seizures (MS + S) exhibit more extensive lesion formation and greater gray matter (GM) atrophy than those without seizures (Calabrese et al., 2008; Wood et al., 2025). Lesions have been observed in both cortical and subcortical regions, including the hippocampus (Nicholas et al., 2016). Histopathological analyses support these imaging findings, revealing significant medial temporal cortex atrophy and a higher burden of GM lesions in MS patients who experience seizures (Calabrese et al., 2008). Together, these studies suggest a strong association between seizure activity in MS and structural damage to gray matter, particularly in regions involved in seizure generation.

Seizures are characterized by abnormal, hypersynchronous neuronal firing resulting from an imbalance between excitatory and inhibitory signaling in the brain (Stafstrom and Carmant, 2015). The primary neurotransmitters regulating this balance are glutamate, which mediates excitation, and GABA, which mediates inhibition. Elevated glutamate levels have been consistently observed in epileptic brain tissue and similarly reported in MS patients (Stover et al., 1997; Sarchielli et al., 2003; Srinivasan et al., 2005). Previous studies have explored alterations in glutamate and GABA-related transporters, receptors, and metabolic enzymes that may contribute to glutamate accumulation in MS brains. However, there is limited research specifically examining these glutamate-related changes in MS patients who also experience seizures. Our lab has previously reported reduced expression of the synaptic glutamate transporter EAAT2 (GLT1) and astrocyte water channel aquaporin-4 (AQP4) in the CA1 region of the hippocampus in MS patients with seizures compared to those without (Lapato et al., 2020). However, the precise mechanisms that predispose only a subset of MS patients to develop seizures remain largely unclear.

The cuprizone (bis-cyclohexanone-oxalyldihydrazone; CPZ) diet is a well-established model of MS (Matsushima and Morell, 2001) and has been shown to induce chronic demyelination-associated seizures (Hoffmann et al., 2008; Lapato et al., 2017). Our previous work demonstrated that 12-week CPZ treatment leads to significant alterations in hippocampal EEG activity, including changes in alpha, beta, and theta frequency power, which were accompanied by CA1 astrogliosis, disrupted AQP4 expression, pyramidal cell layer thinning, and loss of PV-expressing interneurons (Lapato et al., 2017). In the present study, we conducted a longitudinal investigation to quantify the frequency of demyelination-induced electrographic seizures and hippocampal glutamate levels in this CPZ mouse model of MS. Furthermore, we examined hippocampal GABAergic and glutamatergic system alterations, focusing on synaptic proteins, gene expression, and associated neurotransmitter transporters. Our findings provide deeper insight into the mechanisms underlying chronic demyelination-associated epileptogenesis.

## 2. Methods

### 2.1. Ethics statement

All animal experiments were conducted in compliance with the ARRIVE guidelines and the National Institutes of Health (NIH) standards for the care and use of laboratory animals. All procedures were approved by the Institutional Animal Care and Use Committee (IACUC) at the University of California, Riverside (Animal Welfare Assurance #123). Mice were housed in an AAALAC-accredited facility under a 12-h light/dark cycle and provided standard rodent chow ad libitum.

### 2.2. Mice

C57Bl/6 J mice from Envigo, B6.Cg-Tg(Thy1-YFP)16Jrs/J mice (JAX #003709) and PLP-EGFP mice (a generous gift from Dr. Wendy Macklin, University of Colorado, Denver) that were backcrossed to wild-type C57BL/6 mice for over five generations were used. All mice were bred and housed in the vivarium facilities at the University of California, Riverside. Animals were maintained on a 12-h light/dark cycle with ad libitum access to food and water.

### 2.3. CPZ diet preparation and administration

Custom chow containing 0.2 % cuprizone [oxalic bis(cyclohexylidenehydrazide); Sigma–Aldrich, St. Louis, MO] is prepared by Harlan Teklad (2918, Madison, WI). The cuprizone is thoroughly mixed into milled chow, packed in 0.5 kg vacuum-sealed bags and shipped to us. The bags are stored in −80 °C for long-term preservation. Prior to use, one pouch of cuprizone pellets is equilibrated to room temperature for at least 24 h. For treatment, three to four pellets per mouse are placed in the cages every other day for 3–12 weeks (Crawford et al., 2009a, 2009b). Demyelination groups are referred to in figures as 3wk CPZ, 6wk CPZ, 9wk CPZ, and 12wk CPZ. A control group of mice were fed normal diet (Picolab, St. Louis, MO). In each experiment, *n* = 4 to 10 mice/group were used.

### 2.4. Electrode preparation

A 3-channel twisted stainless-steel electrode (Plastics One, MS333/3-A/SPC) was used for all electroencephalography (EEG) implantation surgeries(Jonak et al., 2018). The twisted bipolar wires were cut to 2 mm for implantation into the dorsal hippocampus and the untwisted wire was cut to 0.5 mm to ground in the cortex. To ensure high-fidelity EEG recordings, ~0.5 mm of the insulating coat was removed at the distal tip of all wires.

### 2.5. Electrode implantation

Male PLP-EGFP mice treated with CPZ diet (*n* = 8/group) or normal diet (*n* = 6), totaling 30 mice, for 5 weeks underwent electrode implantation surgery. Mice were anesthetized with isoflurane inhalation (0.2–0.5 %) and given ketamine (80 mg/kg, i.p.) (Zoetis, 10004027) and xylazine (10 mg/kg, i.p.) (Bimeda, 1XYL003). Mice were aseptically prepared for surgery and secured in a stereotaxic apparatus. Artificial tear ointment was applied to the eyes to prevent drying. A midline sagittal incision was made along the scalp to expose the skull. A cotton-tip applicator was used to remove the periosteum from the skull, and the skull was cleaned with saline. A dental drill was used to drill a 1 mm diameter hole over the right frontal bone. Bone dust was washed off with saline and the dura was carefully detached using a 27 G needle. The electrode was then slowly lowered into the hippocampus (AP = 1.8 mm, ML = +1.6 mm from bregma) until the electrode pedestal rested gently on top of the skull. Dental cement (Kuraray, 3382KA) was applied over the skull surface and surrounding the electrode pedestal. A subcutaneous injection of 0.1 mg/kg buprenorphine (Reckitt & Colman, 5053624) was administered. Mice were placed on a heating pad to aid in recovery from anesthesia. Additional doses of buprenorphine were administered every 6–8 h for continuous analgesia during the first 48 h after surgery. Mice were single housed and remained on their respective diets for the duration of the study.

### 2.6. Video electroencephalography (vEEG) recording

Continuous vEEG acquisition began at the 6-, 9-, and 12-week timepoints for a duration of 7 days. Mice were given access to food and water ad libitum and were freely moving. Behavioral activity was video monitored with camera system (BIOPAC CAMSYS4) and time synchronized with EEG recording. Mice were recorded using a tethered system and were connected to the acquisition system via commutator to allow for freedom of motion. EEG recordings were obtained using a digital acquisition system (BIOPAC MP160, Acknowledge 5 software). EEG output was amplified with a gain of 1000 and a sampling rate of 625 Hz. Spontaneous seizures were defined as spiking epileptiform activity lasting continuously for at least 5 s at a frequency of 3 Hz. Each seizure was manually identified and confirmed by two blinded observers.

### 2.7. Glutamate ELISA

Glutamate concentrations (μmol/mL) from homogenized hippocampus were measured using a colorimetric ELISA kit (Abcam, ab83389) according to the manufacturer’s instructions. 21 total mice from DM (*n* = 3–5/group) and normal (*n* = 5) groups were used. Mice were deeply anesthetized and the whole brain was removed, washed with ice cold saline. The hippocampus was dissected, added in a tube with radioimmunoprecipitation (RIPA) lysis buffer supplemented with a phosphatase inhibitor and protease inhibitor. The hippocampi were homogenized in the lysis buffer cocktail, centrifuged and supernatant was collected. Total protein concentration was determined by using protein assay reagent (BioRad, Hercules, CA). Standards were run in duplicates, and all samples were run in triplicates. The absorbance was read at 450nm using a microplate reader (Bio-Rad Laboratories, Hercules, CA).

### 2.8. Perfusions, tissue preparation, and IHC

Groups of mice (n = 6–8/group) were deeply anesthetized with isoflurane and intracardially perfused with ice-cold PBS followed by 10 % formalin (Fisher Scientific, Hampton, NH). The brain was collected, cryoprotected, sagittal sectioned, and subjected to IHC as previously described (Lapato et al., 2017).

### 2.9. Microscopy, quantification, and statistics

40 μm brain sections (2 sections/mouse) were subjected to IHC and imaged using an Olympus BX61 spinning disk confocal microscope at 10×, 20×, and 40× magnifications. *Z*-stack images were acquired, and projection images were compiled using Slidebook 6 and cellSens software (Intelligent Imaging Innovations Inc., Santa Monica, CA). Antibodies used are in Supplementary Table 1. Immunofluorescence intensity and cell numbers in the CA1 stratum pyramidal (SP) and stratum radiatum (SR) were quantified using NIH ImageJ software (v1. 50i http://rsb.info.nih.gov/ij/). The proportion of the SP and SR occupied by pixels, %Area, were assessed after converting images to grayscale and setting the staining intensity threshold as previously described (Lapato et al., 2017; Lapato et al., 2020). The SP and SR regions were delineated, followed by quantification of the staining intensity divided by total area. Each data point represents an average from 2 sections/brain. For cell density, three 40× images along the CA1 SP were used to count cell bodies and measure the area (mm^2^) of the region of interest. The average number of cells was divided by the average area of the SP. Results were analyzed in GraphPad Prism for statistical significance.

### 2.10. Golgi stain

FD Rapid GolgiStain^™^ kit was used to perform Golgi staining. This method is described in Ramón-Moliner and Glaser and Van der Loos (Du, 2019). Briefly, mice were deeply anesthetized, and the brain was rapidly dissected out of the skull. The brain was washed in double distilled cold water to remove blood from the surface. Then the brain was immersed in an impregnation solution (Solution A/B, proprietary solution in the kit) and stored at room temperature for 2 weeks in the dark. The impregnation solution was replaced after the first 6 h. of immersion. The brains were transferred into Solution C and stored at room temperature in the dark for at least 4–7 days. Solution C was replaced at least once after the first 24 h. Brains were sectioned on to gelatin coated slides and stained with staining Solutions D/E for 10 min, washed and then counterstained with Cresyl violet. The slides were then dehydrated in 50 %, 75 %, 95 %, and 100 % ethanol washes. Immediately after, slides were cleared with xylene and cover slipped with Permount (Fisher Scientific, Hampton, NH) mounting medium.

### 2.11. Sholl analysis

Golgi stained CA1 pyramidal neurons were imaged at 20× magnification using brightfield microscopy with an Olympus BX61 microscope. The magnified Z-stacked images (50–75 images, 0.79um apart) were then transferred onto ImageJ where a minimum intensity projection was created, and neurons were traced manually on the Z-projection while using the Z-stack as a guide. Sholl analysis was performed using the plugin for ImageJ that automates the task of doing Sholl analysis on a neuron (Sholl Analysis, Ghosh Lab, UCSD, https://ghoshlab.org/software/ShollAnalysis.pdf). A total of 16 mice were separated into control and DM groups (*n* = 4 mice/group).For analysis, a radius step size of 1 μm was set and 5 pyramidal neuron samples per radius/brain section (3 brain section/mouse) were used to achieve the mean value.

### 2.12. Western blot analysis

Mice were deeply anesthetized and whole brain was removed, washed with ice cold saline. The hippocampus was dissected, added in a tube with RIPA lysis buffer supplemented with a phosphatase inhibitor and protease inhibitor. The tube containing the hippocampus was flash frozen with dry ice and stored at −80 °C. Hippocampi were thawed and homogenized in the lysis buffer cocktail. Supernatants were collected, aliquoted, and stored at −80 °C. Bradford assay was performed to determine protein concentration using the Pierce^™^ Microplate BCA Protein Assay Kit (ThermoFisher Scientific, Rockford, IL). 20 μg of hippocampus protein was boiled in 4× Laemmli buffer with SDS (ThermoFisher Scientific, Ward Hill, MA) at 70 °C for 10 min. Samples and a standard (Precision Plus Protein Dual Color Standards; BIORAD, USA) were run on a 4–20 % precast polyacrylamide gel (BIO-RAD, USA) then transferred to a PVDF membrane (BIO-RAD, USA) for 18.5 h at 4 °C at 30 V. Following transfer, membranes were blocked in a solution of 1× Tris buffered saline (1× TBS; 50 mM Tris–HCl pH 7.4 and 150 mM NaCl) and 5 % powder milk. Membranes were incubated with various primary antibodies overnight at 4 °C in blocking buffer containing 1× TBS with 0.1 % Tween 20 (TBST) and 5 % powder milk. Monoclonal mouse anti-Glyceraldehyde-3-Phosphate Dehydrogenase (EMD Millipore; 1:50,000; 34 kDa) was used as a control for loading errors as well as with other antibodies to assess different levels of proteins. Antibody details are presented in Supplementary Table 1. Primary antibody incubated blots were washed three times with Tris-Buffered Saline with Tween 20 and incubated with relevant horseradish peroxidase-linked secondary antibodies. Membranes were washed three times, incubated in an enhancer reagent (Clarity Western ECL Substrate; BIORAD, USA) for 1 min, and imaged using a ChemiDoc (BIORAD, USA). Glyceraldehyde phosphate dehydrogenase (GAPDH) band density was compared to different protein bands of interest and quantified. Each lane in the gel represents a hippocampus sample from an individual mouse and quantification was performed using data from *n* = 3–5 mice/group.

### 2.13. RNA extraction

Mice were deeply anesthetized and decapitated. Brain followed by hippocampus was dissected and immersed in 100 μL of cold RIPA buffer and frozen temporarily at −20 °C. Zirconium Oxide microbeads were added to each tube and sample homogenized using a Bullet Blender set at a speed of “9” for 5 min. RLT Plus solution from Qiagen RNeasy Plus Micro Kit (Qiagen, Hilden, Germany) was added and the mixture rehomogenized two more times. Immediately after, RNA isolation was performed using the Qiagen RNeasy Plus Micro Kit according to manufacturer’s protocol. RNA concentration and purity was determined using a Nanodrop spectrophotometer, then samples were analyzed using Agilent 2100 Bioanalyzer/Advanced Analytics Fragment Analyzer. RIN values above 7 were adequate NanoString profiling.

### 2.14. NanoString nCounter gene expression assay

Hippocampus RNA (150 μL/50 ng of RNA per sample) from individual mice were used to perform nCounter gene expression assays (NanoString Technologies, Seattle, WA) using the nCounter Mouse Glial panel (770 genes) according to manufacturer’s instructions. Briefly, 50 ng of unamplified RNA was hybridized with the reporter code set at 65 °C for 18 h. Samples were spun down, nuclease free water was added to the samples, and then the samples were loaded into the nCounter cartridge. The cartridge was run on the NanoString nCounter SPRINT Profiler. Data was exported and analyzed using NanoString nSolver and Advanced Analysis Software. Normalized linear counts for all genes in the panel were used in fold change analysis of control, 6- and 12- week CPZ hippocampus genes. Gene information, raw data, and fold changes are summarized in Supplementary Tables 2 A, 2B, and 3. A total of 12 mice were separated into control (*n* = 6), 6wk CPZ (n = 3), and 12wk CPZ (n = 3) groups.

### 2.15. Statistics

All experiments were repeated in their entirety at least twice, with the exception of the Nanostring RNA assays. For IHC, two sections per mouse were taken for each area of interest in the brain. There were 6–8 mice per group. The Bonferroni post hoc test was used to limit the possibility of finding false positives. IHC data were analyzed by student’s *t*-test or ordinary one-way ANOVA with Bonferroni’s multiple comparisons test. Glutamate levels were analyzed using ordinary one-way ANOVA with Uncorrected Fisher’s LSD multiple comparisons as we had lower number of 12 week CPZ PLP_EGFP mice (had to consistently euthanize the mice due to increased seizure activity). Western data and IHC data were analyzed using ordinary one-way ANOVA with Bonferroni’s multiple comparisons test. Sholl Analysis was performed using ordinary two-way ANOVA with Bonferroni’s multiple comparisons test. For nCounter gene expression analysis, normalized counts were used to calculate Log2 fold changes. Only in the gene expression analysis, demyelination groups were compared to normal using one-way ANOVA and to each other by student *t*-test. Differences between 6wk CPZ and 12wk CPZ were represented by the # symbol. Statistical analysis was performed in GraphPad Prism 10 software using one-tailed student’s *t*-test. All experiments were performed at least twice. Differences were considered significant at the *p < 0.05, **p < 0.01, ***p < 0.001, ****p < 0.0001 level.

## 3. Results

### 3.1. The 6- to 12-week CPZ diet results in progressive hippocampal demyelination

**Fig. 1.**
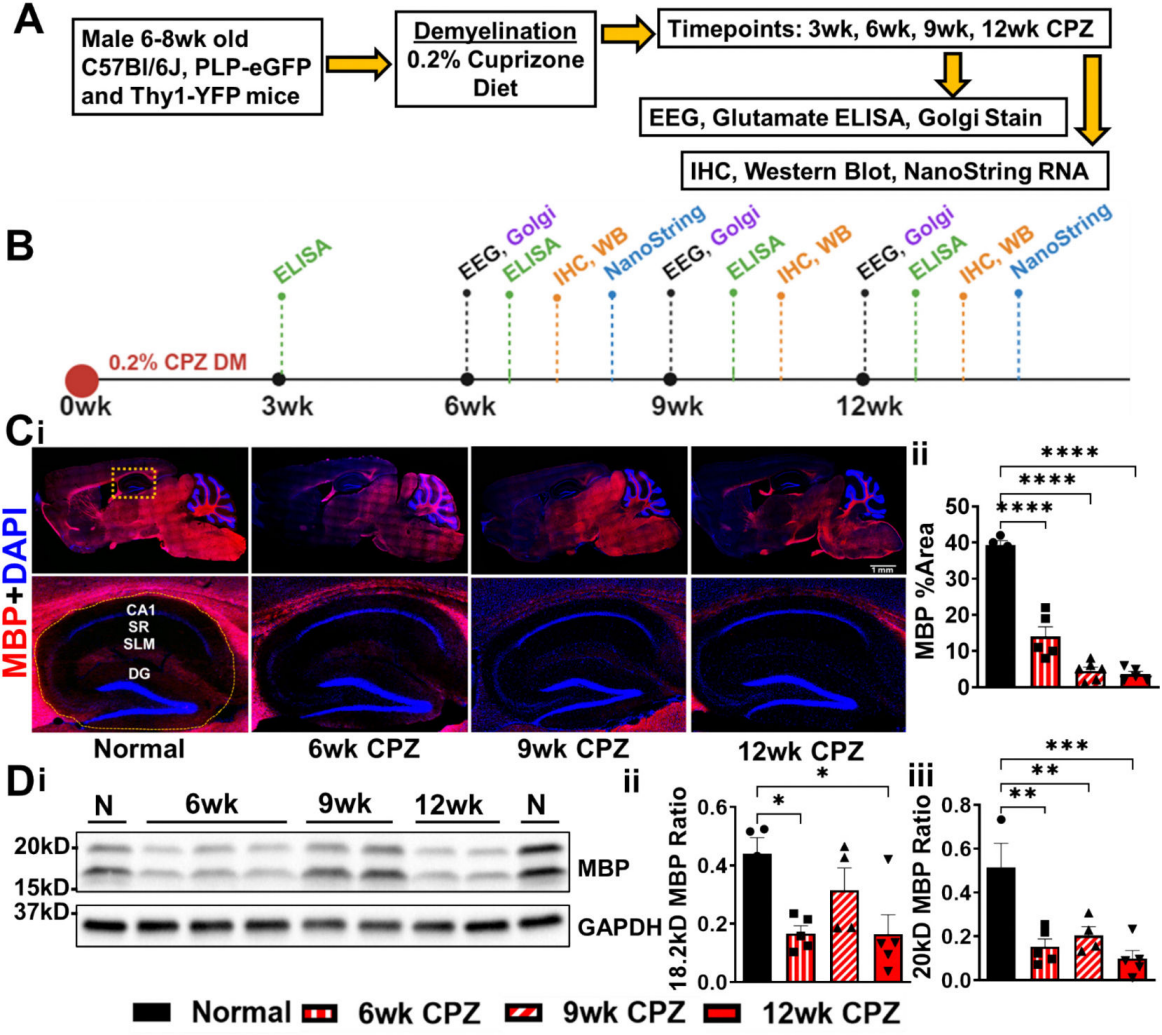
Experimental design and assessment of hippocampal demyelination and seizure activity in the CPZ model. (A) Schematic of the experimental timeline for longitudinal analysis. Male C57Bl/6 J, PLP-eGFP and Thy1-YFP mice (6–8 weeks old) were fed 0.2 % CPZ-milled chow for 6, 9, or 12 weeks. At each timepoint, seizure activity was assessed via intrahippocampal electroencephalogram (EEG) recordings (n = 6–8 mice/group). Following EEG, hippocampal tissue was collected for glutamate quantification by ELISA and protein expression analysis by Western blot (WB). To examine dendritic morphology of CA1 projection neurons, separate cohorts (*n* = 4 mice/group) were euthanized for Golgi staining and Sholl analysis. Additional groups (n = 6 mice/group) were used for immunohistochemistry (IHC). To evaluate transcriptional changes, hippocampal RNA was isolated at 6 and 12 weeks (*n* = 3–6 mice/group) and analyzed using the NanoString Glial Profiling Panel. (B) Timeline indicating which assessments were performed at each timepoint. A 3-week CPZ group (*n* = 5) was included as an acute demyelination reference for glutamate ELISA. (C) Whole Brain and Hippocampus IHC. (i, top) Representative 10× sagittal brain images immunostained for MBP (red) and DAPI (blue) reveal progressive myelin loss in the corpus callosum, hippocampus, cerebellum, and other major white matter tracts. (i, bottom) Higher-magnification image of the hippocampus (orange box) shows defined hippocampal layers: CA1 stratum pyramidal (SP), stratum radiatum (SR), stratum lacunosum moleculare (SLM), and dentate gyrus (DG). (Cii) Quantification of hippocampus MBP fluorescence intensity (demarcated by yellow dashed lines) confirms significant hippocampal demyelination at all CPZ timepoints compared to control. (D) Western blot MBP protein analysis: Hippocampal lysates (i) from control, 6-, 9-, and 12-week CPZ-treated mice were probed for MBP and GAPDH (loading control). (ii) Densitometric analysis of the 18.2 kDa MBP isoform reveals reduced expression at 6 and 12 weeks. (iii) The 20 kDa isoform is significantly decreased at all CPZ timepoints. Data represents *n* = 4 mice/group. as All graphs show mean ± SEM. Statistical significance determined using ordinary one-way ANOVA with Bonferroni’s post hoc test. *p < 0.05, **p < 0.01, ***p < 0.001. Fig. 1B was Created in BioRender. Anderson (2025) https://BioRender.com/27mgyb4

Hippocampal demyelination is a hallmark of both MS and CPZ animal model of MS. In this longitudinal study, we examined the progression of hippocampal demyelination and its association with seizure activity in CPZ-fed mice from 6 to 12 weeks. Male C57BL/6 J, transgenic PLP-EGFP and Thy1-YFP mice were fed a 0.2 % CPZ diet (Crawford et al., 2009b; Lapato et al., 2017) and subjected to longitudinal EEG monitoring for seizure activity (Fig. 1A, B).

At each time point (6, 9, and 12 weeks), subsets of animals were euthanized for tissue collection. Brains were perfusion-fixed, sagittally sectioned, and processed for immunohistochemistry (IHC). In parallel, hippocampal RNA was freshly isolated and analyzed via NanoString nCounter for neuropathology-related gene expression at 6 and 12 weeks.

Immunostaining of sagittal brain sections for myelin basic protein (MBP, red) and DAPI (blue) revealed progressive demyelination in major white matter tracts, culminating in minimal visible immunostaining by 12 weeks (Fig. 1 Ci, top). Notably, increased demyelination in the hippocampus particularly within the stratum lacunosum moleculare (SLM) was observed over time (Fig. 1 Ci, bottom). Quantitative analysis showed a significant reduction in MBP intensity at all time points compared to normal diet controls (F(3,16) = 89.94; 6wk: *p* < 0.0001, 9wk: p < 0.0001, 12wk: p < 0.0001) (Fig. 1Cii). Western blot analysis further confirmed progressive hippocampal demyelination, with significant decreases in MBP isoforms at 18.2 kD (F(3,14) = 5.186; 6wk: *p* = 0.0146, 12wk: *p* = 0.0137) and 20 kD (F(3,13) = 10.90; 6wk: *p* = 0.0011, 9wk: *p* = 0.0056, 12wk: *p* = 0.0003) (Fig. 1Di–iii).

These findings confirm that hippocampal demyelination in the CPZ model progresses over time and aligns with the onset of seizure activity, supporting a mechanistic link between chronic demyelination and epileptogenesis in MS.

### 3.2. Progressive increase in hippocampal seizure activity following sustained CPZ exposure

**Fig. 2.**
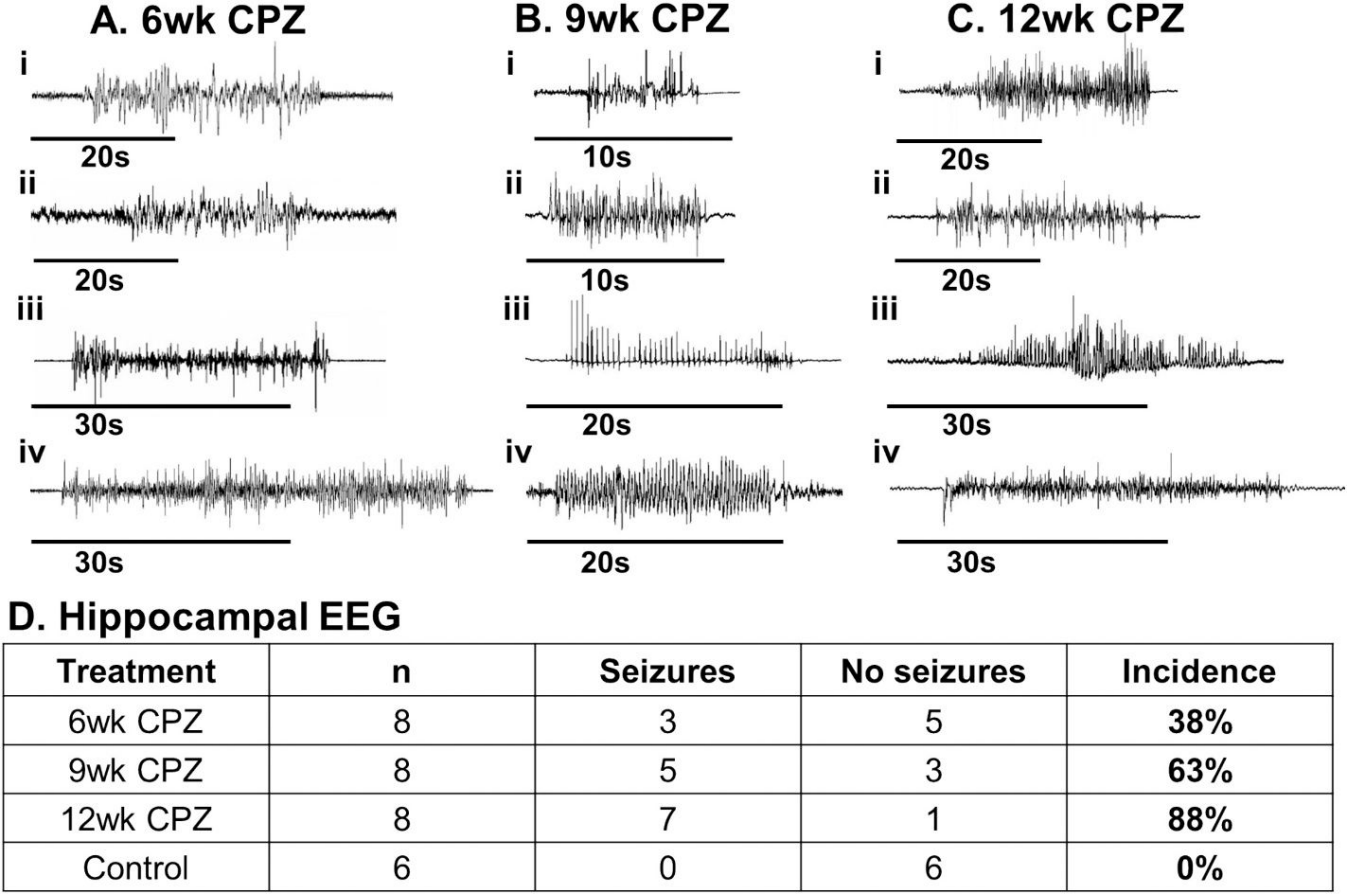
Intrahippocampal EEG recordings reveal progressive seizure activity in chronically demyelinated CPZ-treated mice. (A–C) A 0.2 % CPZ diet was initiated in 6–8-week-old male PLP-EGFP mice on a C57BL/6 background. Groups of mice were surgically implanted with intrahippocampal EEG electrodes. Continuous EEG recordings were conducted during the 6th, 9th, and 12th weeks of CPZ exposure (*n* = 8 mice/group), as well as in age-matched control mice maintained on a standard diet (n = 6 mice). Representative hippocampal EEG traces from CPZ-treated mice at 6 weeks (Ai–iv), 9 weeks (Bi–iv), and 12 weeks (Ci–iv) display spontaneous electrographic seizures of varying duration and morphology, characteristic of abnormal hippocampal network activity. (D) Quantification of seizure incidence reveals a progressive increase in seizure frequency with the duration of CPZ treatment. By 12 weeks, nearly 90 % of CPZ-treated mice exhibited hippocampal seizures, compared to lower rates at 6 and 9 weeks, and no seizures in control mice. These findings indicate a strong correlation between chronic demyelination and heightened seizure susceptibility in the hippocampus.

We and others have previously reported abnormal behavioral activity in chronically CPZ-demyelinated mice compared to those maintained on a normal diet (Franco-Pons et al., 2007; Hoffmann et al., 2008; Lapato et al., 2017). In our earlier work (Lapato et al., 2017), we observed overt tonic-clonic seizures in CPZ-treated mice as early as 8 weeks, along with significant alterations in EEG waveforms and frequency band power between 9 and 12 weeks. However, seizures can also present in more subtle forms, such as absence seizures, which are characterized by brief periods of behavioral arrest or unresponsiveness (Panayiotopoulos et al., 1992; Agathonikou et al., 1998; Tan et al., 2007). MS-associated seizures may also be focal without secondary generalization (Dagiasi et al., 2018; Catenoix et al., 2025). To objectively track the progression of seizure activity with advancing demyelination, we performed intrahippocampal EEG recordings at 6, 9, and 12 weeks of CPZ exposure using an indwelling 3-channel recording electrode. These recordings enabled the detection of spontaneous electrographic seizures across timepoints. Representative EEG traces from CPZ-treated mice revealed spontaneous hippocampal seizures of varying duration, with increasing frequency over time (Fig. 2A–C). Notably, the incidence of electrographic seizures rose markedly with disease progression: 38 % of mice displayed seizure activity at 6 weeks, increasing to 63 % at 9 weeks, and reaching 88 % by 12 weeks (Fig. 2D). These results provide the first in vivo evidence of a progressive increase in hippocampal seizure incidence that correlates with the extent of chronic demyelination.

### 3.3. Thinning of the CA1 pyramidal layer resulting from reduced numbers of PV+ interneurons and diminished dendritic arborization

**Fig. 3.**
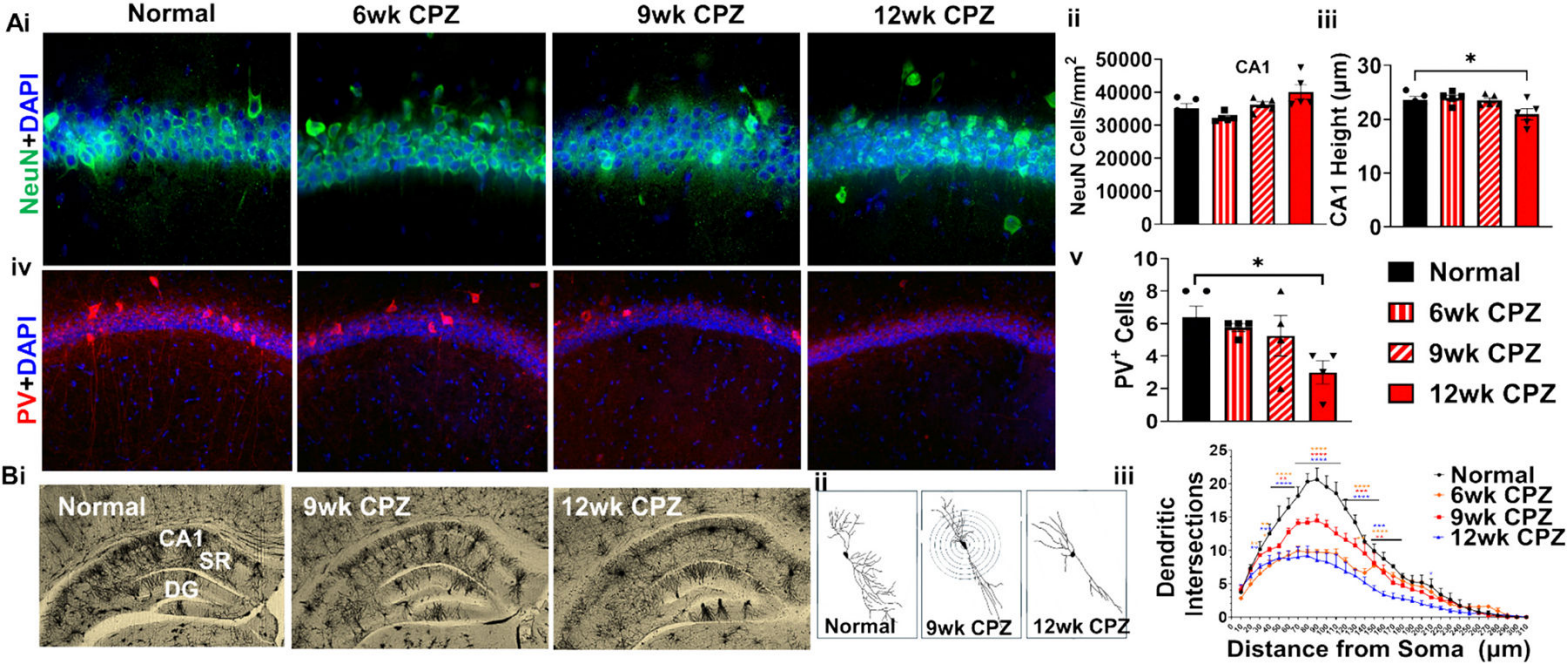
Atrophy of the CA1 pyramidal layer in the demyelinated hippocampus is associated with reduced PV+ interneurons and diminished dendritic arborization of projection neurons. (A) To examine structural alterations in the CA1 pyramidal layer following demyelination, hippocampal sections were immunostained and analyzed. (i) IHC was performed using antibodies against NeuN (green) to label neuronal nuclei and counterstained with DAPI (blue), imaged at 40× magnification. (ii) Quantification revealed no change in NeuN+ cells during CPZ exposure. (iii) A significant reduction in the total thickness of the CA1 pyramidal layer was observed in 12 weeks of CPZ treatment. (iv) Representative 20× images show PV+ interneurons (green) in the CA1 and stratum radiatum (SR), with DAPI (blue) marking nuclei. (v) Quantification demonstrated a significant decrease in the number of PV+ interneurons in the SP region of CA1 in 12 weeks CPZ groups as compared to controls. (B) Dendritic complexity of CA1 projection neurons was assessed via Golgi staining followed by Sholl analysis. (i) Representative 2× magnification images of Golgi-stained coronal hippocampal sections showing the CA1, SR, and dentate gyrus (DG). (ii) Example tracings of individual pyramidal neurons used for Sholl analysis. (iii) Sholl analysis revealed a significant reduction in dendritic intersections in all CP*Z*-treated groups compared to controls. Data represent *n* = 4–6 mice/group for all analyses; Sholl analysis included 5 neurons/mouse/group. All graphs show mean ± SEM. Statistical significance determined using ordinary one-way ANOVA (A) and two-way ANOVA (B) with Bonferroni’s multiple comparisons test. *p < 0.05, **p < 0.01, ***p < 0.001, ****p < 0.0001.

Neuronal cell bodies stained for NeuN (green) within the CA1 pyramidal layer were co-labeled with nuclear dye DAPI (blue) and imaged at 40× magnification (Fig. 3Ai). Quantification of NeuN+ cells revealed no significant differences between control and CPZ treated group (Fig. 3Aii). Structural atrophy of the CA1 region was observed only at 12 weeks, as indicated by a significant reduction in the thickness of the CA1 pyramidal layer (F(3,16) = 4.246, *p* = 0.0377) (Fig. 3). To evaluate the status of PV expression that could indicate changes in PV+ inhibitory interneurons, brain sections were immunostained and imaged in the dorsal hippocampus at 20× magnification (Fig. 3Aiv). A marked decline in PV+ cells (red) was observed in the 12-week CPZ group, with approximately a 50 % reduction relative to normal diet animals (F(3,13) = 3.468, *p* = 0.0252) (Fig. 3Av).

Longitudinal morphological changes in CA1 projection neurons during demyelination were assessed using Golgi staining (Fig. 3Bi) followed by Sholl analysis. Individual CA1 pyramidal neurons were manually reconstructed using the Simple Neurite Tracer plugin in ImageJ (Fig. 3Bii), and dendritic complexity was quantified via Sholl analysis by counting intersections at increasing distances from the soma using an ImageJ plugin (Ferreira et al., 2014). Reduced dendritic arborization was detected in the 6- and 12-week CPZ groups at 30 μm from the soma, with all CPZ-treated groups showing significant decreases in dendritic branching from 50 to 130 μm. This reduced complexity extended up to 140 μm in the 6- and 9-week groups and reached as far as 210 μm in the 12-week group (highest-order interaction: F(90, 339) = 5.425, *p* < 0.0001) (Fig. 3Biii). Together, these results demonstrate early cellular responses to demyelination, followed by structural degeneration marked by CA1 atrophy, loss of PV+ interneurons, and progressive disruption of dendritic architecture in pyramidal neurons, highlighting the profound cumulative impact of chronic demyelination on hippocampal integrity.

### 3.4. Alterations in whole hippocampal glutamate levels and expression of glutamate transporters and metabolic enzymes

**Fig. 4.**
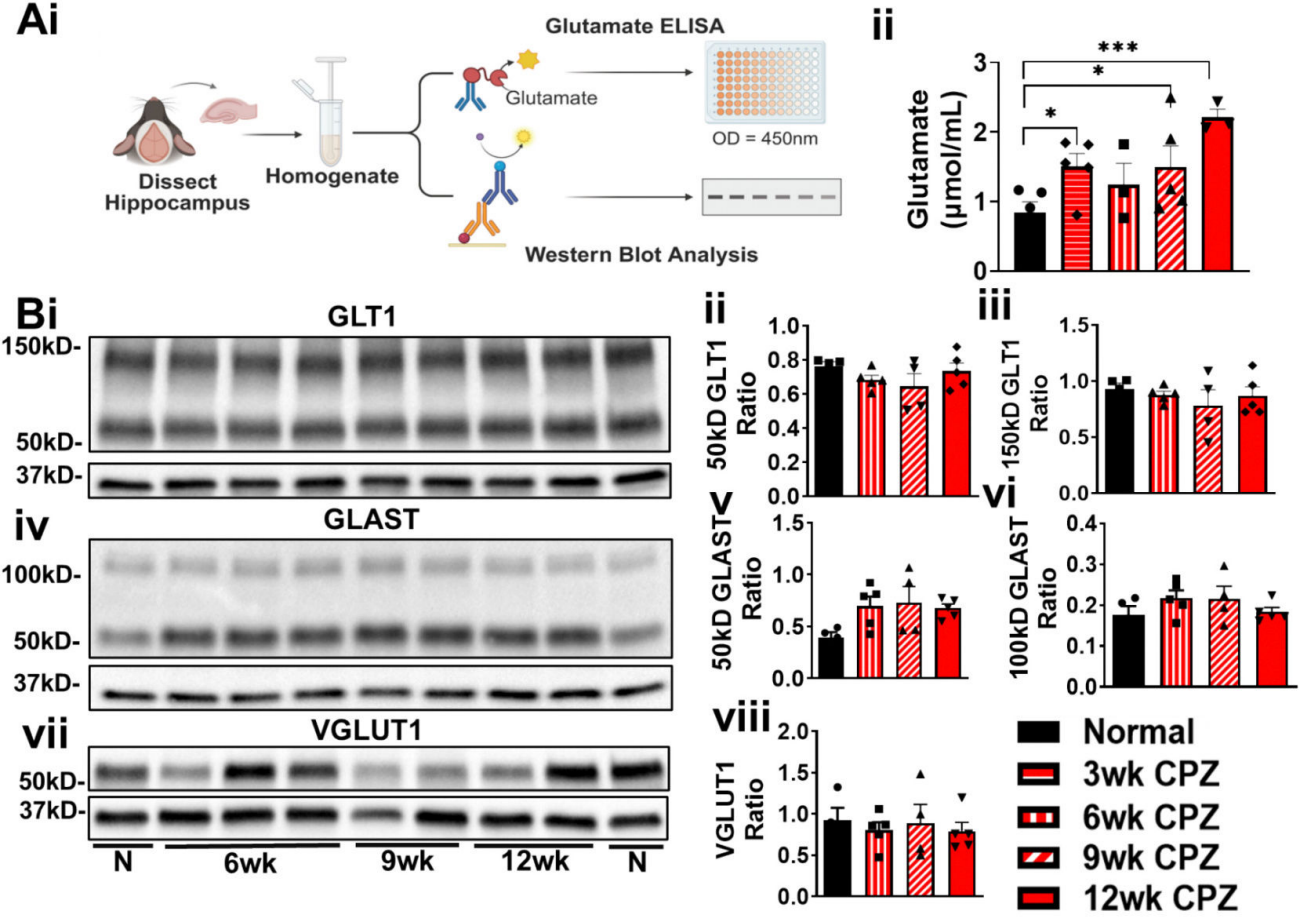
Longitudinal assessment of hippocampal glutamate levels and expression of key glutamatergic transporters during chronic demyelination. (A) To evaluate longitudinal changes in glutamate levels and expression of key glutamatergic transporters in the hippocampus, whole hippocampal tissue was homogenized and analyzed via enzymatic glutamate detection and Western blot (i). Mice fed CPZ diet for 3–12 weeks exhibited significantly increased hippocampal glutamate concentrations at 3, 9, and 12 timepoints compared to controls (ii). Data represent n = 3–5 mice per group. All graphs display mean ± SEM. Statistical comparisons were performed using ordinary one-way ANOVA with Uncorrected Fisher’s LSD multiple comparisons test. *p < 0.05, **p < 0.01, ***p < 0.001. (B) Protein expression of major glutamate transporters—glutamate transporter 1 (GLT1) (i), glutamate aspartate transporter (GLAST) (iv), and vesicular glutamate transporter 1 (VGLUT1) (vii) was assessed by Western blot. Quantification revealed no significant changes in GLT1 (ii, iii), GLAST (v, vi), or VGLUT1 (viii) protein levels across CPZ-treated and control groups. Of note, the unglycosylated forms of GLAST typically appear as bands between 50 and 55 kDa and the 100 kDa band potentially representing multimers. In contrast, GLAST expression was significantly increased at 6 and 12 weeks of CPZ treatment compared to controls (v, vi). Data represent n = 4–6 mice per group. All graphs display mean ± SEM. Statistical comparisons were performed using ordinary one-way ANOVA with Bonferroni’s multiple comparisons test. *p < 0.05, **p < 0.01, ***p < 0.001. Fig. 4Ai was created in BioRender. (Anderson, 2025) https://BioRender.com/27mgyb4

During excitotoxicity, glutamate dynamics can be disrupted in several ways: synaptic glutamate levels may spike beyond normal peak concentrations (over 1.1 mM) for a very short time, remain elevated for prolonged periods (prolonged decay time), or the baseline glutamate concentration may increase independently of neuronal activity. Notably, even modest elevations in baseline glutamate (2–5 μM) are sufficient to trigger neuronal injury (Armada-Moreira et al., 2020). To investigate global changes in hippocampal glutamate concentrations in the CPZ model, we performed glutamate ELISA on hippocampal homogenates from mice treated with CPZ for 3, 6, 9, or 12 weeks (Fig. 4Ai). Glutamate levels were significantly increased in 3-, 9-, and 12-week CPZ time points relative to controls (F(4,16) = 4.024; 3wk: *p* = 0.0441, 9wk: *p* = 0.0457, 12wk: *p* = 0.0013) (Fig. 4Aii). Notably, the glutamate concentration observed in 12 week CPZ hippocampal lysates (~2.2 μM) was double that measured in control samples (~0.9 μM) and within the range reported to cause excitotoxicity and neuronal injury.

Feasibly, this dysregulation of glutamate could reflect disruptions in its trafficking between neurons and astrocytes. Astrocytes are key regulators of glutamate metabolism and also contribute to GABA synthesis and uptake, thus influencing both excitatory and inhibitory neurotransmission (Ullian et al., 2004; Ishibashi et al., 2019). Synaptic glutamate reuptake is primarily mediated by the astrocytic transporters EAAT1 (GLAST) and EAAT2 (GLT1), while vesicular glutamate transporters (VGLUTs) facilitate synaptic release (Zhou and Danbolt, 2014).

To assess whether altered expression of glutamate transporters contributes to changes in hippocampal glutamate levels during demyelination, we performed Western blot analysis for GLT1, GLAST, and VGLUT1 in hippocampal homogenates from 6 to 12-week CPZ-treated mice. Although expression levels varied—potentially reflecting differences between mice with and without seizures— overall, GLT1 (Fig. 4Bi–iii), GLAST, and VGLUT1 (Fig. 4Bvii-viii) remained largely unchanged across groups. These findings suggest that hippocampal glutamate homeostasis is disrupted during chronic demyelination, without overall changes in astrocytic and neuronal transporter expression. However, these observations represent the entire hippocampus and lack the specificity to detect regional changes.

### 3.5. Regional alterations in glutamate transporter and enzyme expression within the CA1 region of the demyelinated hippocampus

**Fig. 5.**
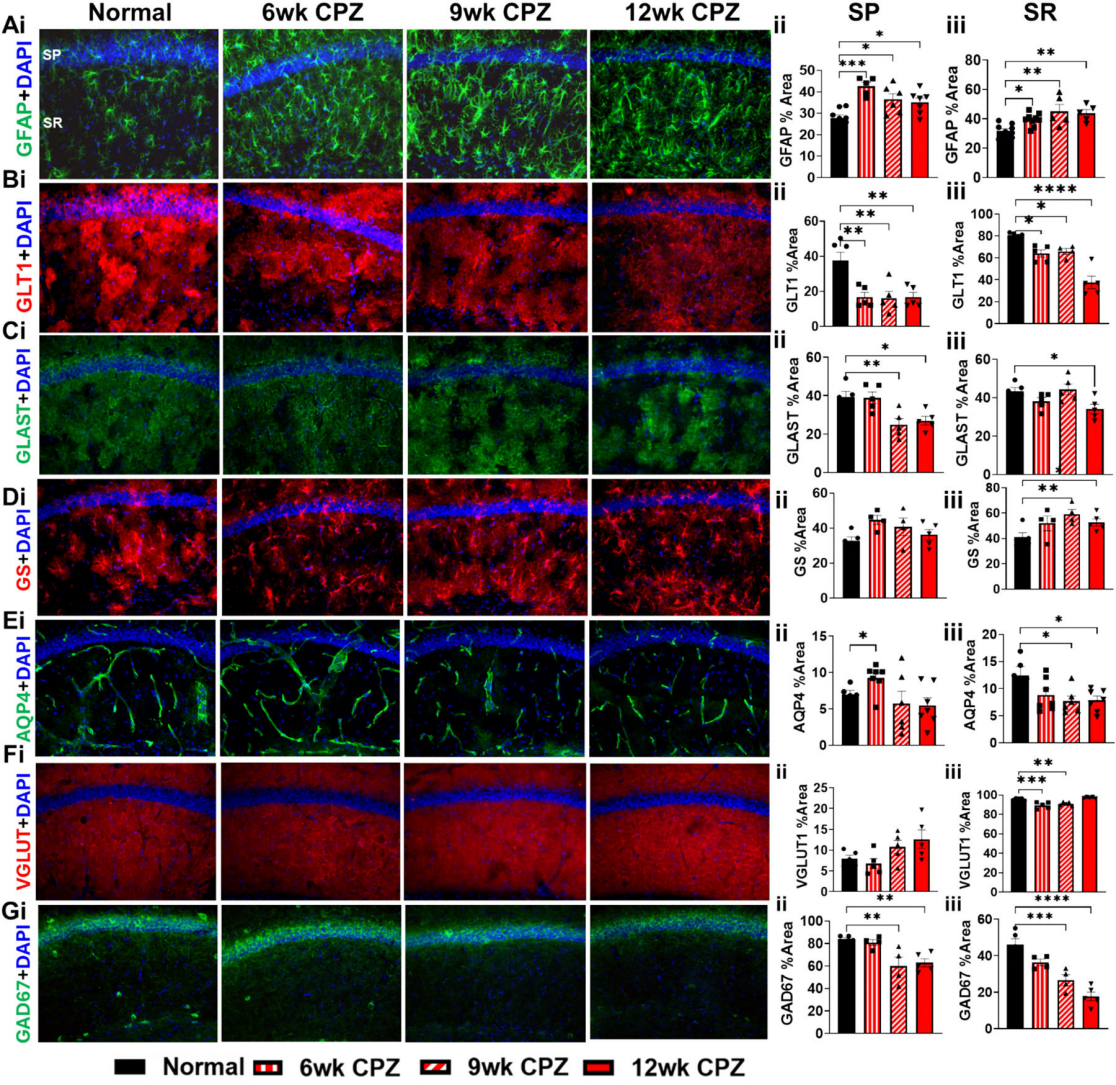
Alterations in Glutamate Transporters and Enzymes in the CA1 Pyramidal Layer During Demyelination. Immunostaining and subsequent analysis were performed to examine astrocyte reactivity and the expression of key glutamate-related transporters and enzymes within the SP and SR of the CA1 region in hippocampus containing brain sections. 20× magnification images of sagittal hippocampal sections are shown in Ai-Gi. These sections were stained for: GFAP (A, green) to visualize astrocytes, astrocyte glutamate transporter, GLT1 (B, red) and GLAST (C, green), an enzyme in astrocytes that converts glutamate to glutamine GS (D, red), glutamine synthetase, an aquaporin associated with astrocytes, AQP4 (E, green), a neuronal vesicular glutamate transporter, VGLUT1 (D, red), and glutamate decarboxylase 67, an enzyme in GABAergic neurons (maybe astrocytes as well) that converts glutamate to GABA (GAD67) (G, green). All were co-stained with DAPI (blue) for cellular visualization. (A-G ii-iii) Quantification in the SP and SR revealed astrogliosis (Aii, iii), indicating increased astrocyte reactivity, with a significant decline in GLT1 (Bii, iii) expression across all time points. GLAST (Cii, iii) decreased during chronic demyelination. Elevated immunoreactivity for GS (Diii) was only observed in the SR at chronic demyelination. AQP4 (Eii, iii) in the SP was higher at 6 weeks CPZ but was decreased in the SR at 9 and 12 weeks CPZ. VGLUT1 (Diii) immunoreactivity in the SR was reduced at 6 and 9 weeks CPZ. GAD67 (Gii, iii) expression was significantly reduced in the SP and SR at chronic demyelination compared to controls. (A-G ii-iii) Quantification in the SP and SR are shown. Data represents n = 4–8 mice per group. as All graphs display mean ± SEM. Statistical analysis was performed using ordinary one-way ANOVA with Bonferroni’s multiple comparison test. *p < 0.05, **p < 0.01, ***p < 0.001, ****p < 0.0001.

To determine whether glutamate transporter expression is regionally altered during demyelination, IHC was performed on sagittal hippocampal sections from 6-, 9-, and 12-week CPZ-treated mice. Astrocytes in the CA1 region were immunolabeled for glial fibrillary acidic protein (GFAP, green) and counterstained with DAPI (blue) (Fig. 5Ai). Across all demyelination time points, astrocytes in the pyramidal layer (SP) exhibited reactive changes, including hypertrophy and increased GFAP expression (F(3,22) = 9.110; 6wk: *p* = 0.0001, 9wk: *p* = 0.0144, 12wk: *p* = 0.0348) (Fig. 5Aii). Similar reactive astrogliosis was observed in the stratum radiatum (SR) (F(3,24) = 7.232; 6wk: *p* = 0.0375, 9wk: *p* = 0.0015, 12wk: *p* = 0.0043) (Fig. 5Aiii).

GLT1 (red) immunoreactivity was significantly reduced in the SP at all CPZ time points (F(3,17) = 8.953; 6wk: *p* = 0.0023, 9wk: *p* = 0.0017, 12wk: p = 0.0023) (Fig. 5Bi–ii). The SR also showed decreased GLT1 expression (F(3,15) = 24.58; 6wk: *p* = 0.0151, 9wk: *p* = 0.0434, 12wk: *p* < 0.0001; Fig. 5Biii). Similarly, GLAST (green) was reduced in the SP at 9- and 12-weeks CPZ (F(3,16) = 7.245; 9wk: *p* = 0.0076, 12wk: *p* = 0.0217) and the SR at only 12 weeks CPZ (F(3,16) = 4.130; 12wk: *p* = 0.0392) (Fig. 5Ci–iii). GS (red), responsible for converting glutamate to glutamine in astrocytes, did not change expression in the SP, but increased in the SR at 9 weeks CPZ (F(3,14) = 3.657; 9wk: *p* = 0.0191) (Fig. 5Di–iii).

To further evaluate astrocyte-related changes, AQP4 (green), an astrocytic water channel involved in potassium and neurotransmitter homeostasis was assessed. AQP4 expression was transiently increased in the SP at 6 weeks (*p* = 0.0219) but significantly decreased in the SR at both 9 and 12 weeks (F(3,21) = 3.317; 9wk: *p* = 0.0324, 12wk: *p* = 0.0344) (Fig. 5Ei–iii). Expression of VGLUT1 (red) did not change in the SP (Fig. 5Di-ii). Conversely, VGLUT1 decreased in the SR at 6 and 9 weeks CPZ (F(3,16) = 18.40; 6wk: *p* = 0.0030, 9wk: *p* = 0.0050), then returned to normal levels at 12 weeks (Fig. 5Fi-iii). GAD67 (green), which catalyzes the conversion of glutamate to the inhibitory neurotransmitter GABA, showed a marked downregulation at 9 and 12 weeks CPZ in both the SP (F(3,14) = 8.182; 9wk: *p* = 0.0044, 12wk: p = 0.0076) and SR (F(3,14) = 22.50; 9wk: *p* = 0.0005, 12wk: p < 0.0001) (Fig. 5Gi–iii).

These findings reveal region-specific disruptions in astrocyte water channels, glutamate transporters, and glutamatergic enzyme expression within the CA1 during chronic demyelination, highlighting a complex reorganization of excitatory and inhibitory neurotransmission machinery in response to sustained myelin loss.

### 3.6. mRNA transcript analysis reveals significant gene expression changes associated with demyelination and astrogliosis in 6- and 12-week CPZ-treated mice

**Fig. 6.**
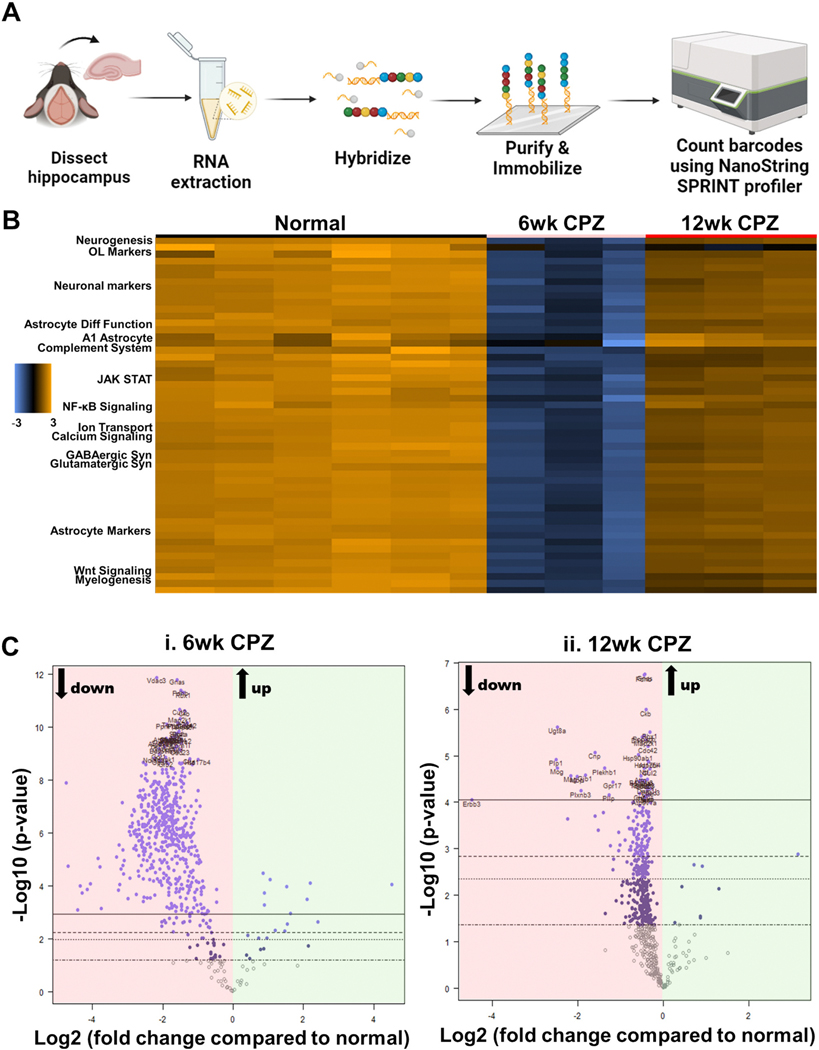
Increased transcriptional alterations in genes of chronically demyelinated hippocampi. (A) Experimental Design: To assess longitudinal changes in the hippocampus under CPZ demyelination, mRNA was extracted from hippocampal homogenates of mice treated with CPZ for 6 and 12 weeks. These samples were then analyzed using the nCounter Glial panel (NanoString Technologies) to quantify gene expression. Fig. 6A was created in BioRender. (Anderson, 2025) https://BioRender.com/27mgyb4. (B) Heat Map of Gene Expression Themes: A heat map displays the expression profiles of key gene themes relevant to the current study, clustered by experimental group. These groups include Normal (left, indicated by a black bar), 6 weeks CPZ (center, indicated by a pink bar), and 12 weeks CPZ (right, indicated by a red bar). Gene group expression is represented as Z-scores, with orange indicating upregulation and blue indicating downregulation. (C) Differential Expression Analysis (Volcano Plots): Volcano plots illustrate the results of differential expression analysis. Panel (Ci) shows the differential gene expression at 6 weeks CPZ compared to normal controls, while panel (Cii) displays the differential gene expression at 12 weeks CPZ compared to normal controls.

To investigate longitudinal molecular alterations in the hippocampus during demyelination, we performed transcriptomic profiling in 6- and 12-week CPZ-treated mice using the NanoString Glial Profiling gene expression panel (Fig. 6A). This panel assays 770 mouse and human genes associated with glial homeostasis, cellular stress, neuroinflammation, and neurotransmission—pathways that are frequently disrupted in both MS and its animal models, such as EAE (Baltan et al., 2021; Weerasinghe-Mudiyanselage et al., 2022).

mRNA from hippocampal homogenates was extracted from normal (*n* = 6), 6-week CPZ (*n* = 3), and 12-week CPZ (n = 3) mice, total of *n* = 12 mice. Full gene expression raw data and fold changes are provided in Supplementary Table 3. Using significance thresholds of *p* < 0.05 and log₂ fold-change, 6-week CPZ mice showed extensive gene repression, with 564 genes downregulated and 21 upregulated. At 12 weeks, 395 genes were downregulated and 8 significantly upregulated compared to controls. *Gjb1* (−6.59, *p* < 0.0001), the connexin 32 gene found on OLs, and *Erbb3* (−4.48, p < 0.0001), which is an early neurodevelopmental gene, are the top downregulated genes at 6 and 12 weeks CPZ, respectively. *Clec7a*, a pattern recognition receptor in microglia, is the top upregulated gene for both 6 weeks (4.51, *p* < 0.0001) and 12 weeks (3.14, *p* = 0.00129) CPZ, suggesting a durable microglial response. A heat map of the genes categorized by themes (Supplementary Table 2B) demonstrate dramatic downregulation of major pathways at 6 weeks CPZ (Fig. 6B). Volcano plots further illustrate differentially expressed genes in both 6- and 12-week groups (Fig. 6 Ci-ii). The top 20 upregulated and downregulated genes for 6- and 12-week CPZ versus normal are summarized in Supplementary Table 4.

**Fig. 7.**
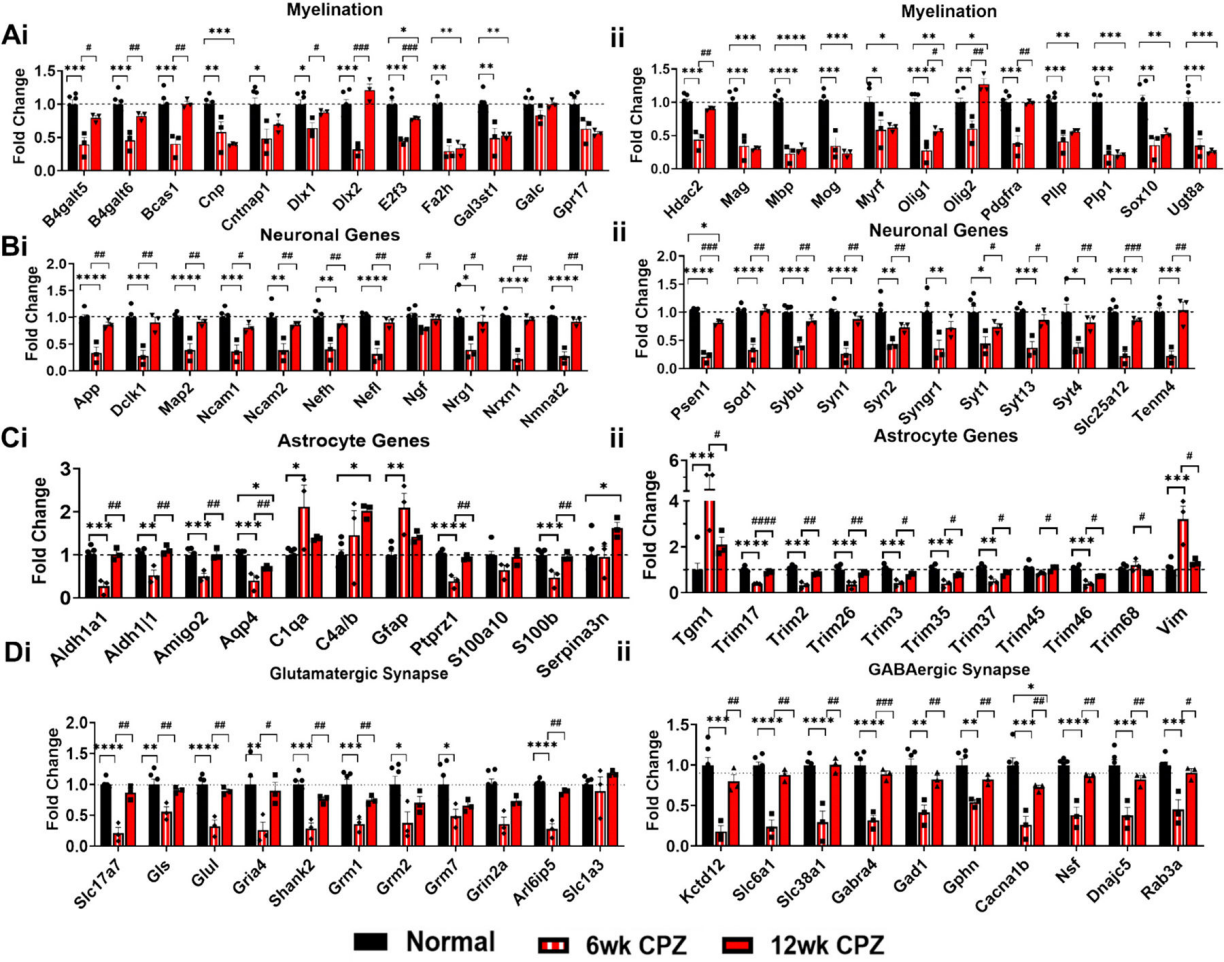
Glia and Neuronal-specific gene modifications during CPZ-induced hippocampal demyelination. (A-D) Fold change quantification of selected genes related to myelin, neurons, astrocytes, and glutamatergic and GABAergic systems in the hippocampus following CPZ demyelination. (Ai, ii) Myelin-related gene changes: Significant decreases in myelin-related gene expression were observed during 6 and 12 weeks of CPZ treatment. *B4galt5, B4galt6, Bcas1, Dlx1, Dlx2, E2f3, Hdac2, Olig1, Olig2,* and P*dgfra* showed increased expression between 6 and 12 weeks of CPZ. Notably, *Olig2* expression was significantly increased in 12 weeks CPZ compared to the control group. (Bi, ii) Neuronal genes changes: Most neuron genes responsible for neuronal development, structural integrity, and synaptic neurotransmission demonstrated significant downregulation at 6 weeks CPZ, then returned to near normal levels by 12 weeks CPZ. Interestingly, *Psen1* remained downregulated with demyelination compared to the control group, even though expression increased between 6 and 12 weeks CPZ. (Ci, ii) Astrocyte-related gene changes: These panels show an upregulation of proinflammatory astrocyte genes and a downregulation of neuroprotective astrocyte genes. *Aldh1a1, Aldh1|1, Amigo2, Aqp4, Ptprz1, S100b, Tgm1, Vim*, and the *TRIM* family showed expression levels increased at 12 weeks CPZ compared to 6 weeks CPZ. (Di) Glutamatergic synaptic gene changes: Glutamatergic synaptic genes exhibited significant downregulation at 6 weeks CPZ compared to controls. *Slc17a7, Gls, Glul, Gria4, Shank2, Grm1*, and *Arl6ip5* levels increased at 12 weeks compared to 6 weeks CPZ. Importantly, *Slc1a3* did not change with CPZ demyelination. (Dii) GABAergic synaptic gene changes: GABAergic gene expression was downregulated at 6 weeks CPZ. While most genes showed a transcriptional rebound, *Cacna1b* and *Nsf* remained downregulated at 12 weeks CPZ. All other presented genes in this panel demonstrated significant upregulation at 12 weeks compared to 6 weeks, indicating a potential transcriptional rebound during chronic demyelination. All graphs display mean ± SEM, with *n* = 3–6 mice per group. Fold changes were calculated from normalized expression data, and statistical significance was determined using ordinary one-way ANOVA with Bonferroni’s multiple comparison test. *p < 0.05, **p < 0.01, ***p < 0.001, ****p < 0.0001 represent statistical significance between normal and 6 week CPZ and normal to 12 week CPZ. Statistical significance between 6- and 12-week CPZ was determined using unpaired *t*-test and represented by: #p < 0.05, ##p < 0.01, ###p < 0.001, ####p < 0.0001.

Overall, significant downregulation of oligodendrocyte/myelin-related genes was found during demyelination. These include *Cnp* (F(2,9) = 16.53; 6wk: *p* = 0.0094, 12wk: *p* = 0.0009)*, E2f3* (F(2,9) = 26.17; 6wk: *p* = 0.0001, 12wk: *p* = 0.0374)*, Fa2h* (F(2,9) = 19.68; 6wk: *p* = 0.0010, 12wk: *p* = 0.0016)*, Gal3st1* (F(2,9) = 13.49; 6wk: *p* = 0.0035, 12wk: *p* = 0.0055)*, Olig1* (F(2,9) = 34.79; 6wk: p < 0.0001, 12wk: *p* = 0.0021)*, Pllp* (F(2,9) = 26.76; 6wk: *p* = 0.0002, 12wk: *p* = 0.0015)*, Sox10* (F(2,9) = 10.49; 6wk: *p* = 0.0046, 12wk: *p* = 0.0246)*,* and *Ugt8a* (F(2,9) = 33.27; 6wk: *p* = 0.0003, 12wk: p = 0.0001) which are all associated with OPC differentiation to mature OLs and myelin formation. Genes responsible for myelin maintenance and stability like *Mag* (F(2,9) = 27.81; 6wk: *p* = 0.0005, 12wk: p = 0.0003), *Mbp* (F(2,9) = 49.06; 6wk: *p* < 0.0001, 12wk: p < 0.0001), *Mog* (F(2,9) = 29.67; 6wk: *p* = 0.0006, 12wk: p = 0.0002), *Myrf* (F(2,9) = 6.532; 6wk: *p* = 0.0266, 12wk: *p* = 0.0401), and *Plp1* (F(2,9) = 30.99; 6wk: p = 0.0002, 12wk: p = 0.0002) were also significantly downregulated. Interestingly, the OL lineage marker *Olig2* was downregulated at 6 weeks (*p* = 0.0068) but upregulated at 12 weeks compared to both control (*p* = 0.0196) and 6-week CPZ (*p* = 0.0047). Similarly, other OL lineage and early myelin formation genes, *B4galt5* (*p* = 0.0109), *B4galt6* (*p* = 0.0078), *Bcas1* (*p* = 0.0027), *Dlx1* (*p* = 0.0244), *Dlx2* (p = 0.0006), *Hdac2* (*p* = 0.0028), *Olig1* (p = 0.02), *and Pdgfra* (*p* = 0.0033) showed increased expression between 6- and 12-week CPZ, possibly indicating an attempt at remyelination or transcriptional rebound (Fig. 7Ai-ii). *Galc,* a galactosylceramidase, and *Gpr17,* a marker for a subset of oligodendrocyte precursors, did not change with demyelination (Fig. 7Ai).

Neuron-associated genes exhibited a consistent pattern of downregulation at 6 weeks of CPZ treatment, followed by significant recovery by 12 weeks (Fig. 7B). This group includes *Dclk1* (F(2,9) = 19.90; 6wk: *p* = 0.0003; 12wk: *p* = 0.0061), *Ncam1* (F(2,9) = 17.04; 6wk: p = 0.0005; 12wk: *p* = 0.0142), *Ncam2* (F(2,9) = 12.85; 6wk: *p* = 0.0014; 12wk: *p* = 0.0098), *Nrg1* (F(2,9) = 5.000; 6wk: *p* = 0.0256; 12wk: *p* = 0.0171), *Nrxn1* (F(2,9) = 52.16; 6wk: *p* < 0.0001; 12wk: *p* = 0.0010), *Syn1* (F (2,9) = 30.90; 6wk: p < 0.0001; 12wk: p = 0.0028), *Syn2* (F(2,9) = 8.958; 6wk: p = 0.0047; 12wk: *p* = 0.0076), and *Tenm4* (F(2,9) = 17.06; 6wk: *p* = 0.0008; 12wk: *p* = 0.0046), which are critical for neuronal development and synaptogenesis. Genes involved in maintaining neuronal structure, axonal integrity, neurotransmitter release, and synaptic plasticity—such as *App* (F(2,9) = 26.87; 6wk: p < 0.0001; 12wk: *p* = 0.0062), *Map2* (F(2,9) = 27.33; 6wk: p < 0.0001; 12wk: *p* = 0.0080), *Nefh* (F(2,9) = 10.17; 6wk: *p* = 0.0032; 12wk: *p* = 0.0088), *Nefl* (F(2,9) = 38.88; 6wk: p < 0.0001; 12wk: *p* = 0.0038), *Nmnat2* (F(2,9) = 47.66; 6wk: p < 0.0001; 12wk: *p* = 0.0013), *Sod1* (F(2,9) = 34.04; 6wk: p < 0.0001; 12wk: *p* = 0.0015), *Sybu* (F(2,9) = 27.95; 6wk: p < 0.0001; 12wk: p = 0.0028), *Syngr1* (F(2,9) = 6.970; 6wk: *p* = 0.0097; 12wk: *p* = 0.0595), *Syt1* (F(2,9) = 6.110; 6wk: *p* = 0.0143; 12wk: *p* = 0.0479), *Syt13* (F(2,9) = 19.43; 6wk: *p* = 0.0003; 12wk: *p* = 0.0125), *Syt4* (F(2,9) = 4.732; 6wk: *p* = 0.0249; 12wk: p = 0.0088), *and Slc25a12* (F(2,9) = 35.58; 6wk: *p* < 0.0001; 12wk: p = 0.0008) also followed a similar trajectory. In contrast, nerve growth factor (*Ngf*) expression remained unchanged at both 6 and 12 weeks compared to normal but was significantly elevated at 12 weeks relative to 6 weeks (*p* = 0.0106; Fig. 7Bi). Notably, *Psen1*, which plays a key role in neuronal development, remained downregulated in both CPZ groups compared to normal (F(2,9) = 90.75; 6 wk.: *p* < 0.0001; 12 wk.: *p* = 0.0223). However, its expression showed a significant increase in 12 weeks compared to 6 weeks (p = 0.0003; Fig. 7Bii).

Several astrocyte-associated genes were significantly upregulated during CPZ-induced demyelination, including *C1qa* (F(2,9) = 6.793; 6wk: *p* = 0.0101), *C4a/b* (F(2,9) = 12wk: *p* = 0.0432), *Gfap* (F(2,9) = 12.17; 6wk: *p* = 0.0016), *Serpina3n* (*p* = 0.0188), *Tgm1* (F(2,9) = 21.55; 6wk: *p* = 0.0004), and *Vim* (F(2,9) = 17.81; 6wk: *p* = 0.0005) (Fig. 7 Ci-Cii). These genes are markers of reactive astrogliosis, complement activation, and neuroinflammatory signaling (Satoh et al., 2004; Ingram et al., 2010; Xu et al., 2018). Notably, *Tgm1* (*p* = 0.0307) and *Vim* (*p* = 0.0154) expression significantly declined at 12 weeks compared to 6 weeks, indicating a temporal shift in astrocytic activation (Fig. 7Cii). In contrast, several astrocyte-enriched genes involved in water transport, amyloid processing, and cytoskeletal stability were downregulated during 6wk CPZ and recovered at 12wk CPZ. These included *Aldh1a1* (F (2,9) = 17.72; 6wk: *p* = 0.0007) and *Aldh1|1* (F(2,9) = 14.40; 6wk: *p* = 0.0026), *Amigo2* (F(2,9) = 16.37; 6wk: *p* = 0.0009), *Aqp4* (F(2,9) = 20.53; 6wk: p = 0.0003, 12wk: *p* = 0.0236), *Ptprz1* (F(2,9) = 37.70; 6wk: *p* < 0.0001), and *S100b* (F(2,9) = 17.85; 6wk: p = 0.0004) (Fig. 7 Ci), all of which are commonly implicated in neurodegenerative processes (Wolfe et al., 1999; Forrest et al., 2023). Members of the tripartite motif (TRIM) family, whom are involved in regulating many cellular functions especially inflammation, were also found to be significantly downregulated at 6 weeks CPZ – except for *Trim45* and *Trim68* (Fig. 7Cii). Interestingly, *Aqp4* (*p* = 0.0377), *S100b* (*p* = 0.0037), *Trim17* (p < 0.0001), *Trim2* (*p* = 0.0023), *Trim26* (*p* = 0.0059), *Trim3* (*p* = 0.0113), *Trim35* (p = 0.0113), *Trim37* (*p* = 0.0156), and *Trim46* (*p* = 0.0111) were significantly increased at 12 weeks relative to 6 weeks, suggesting dynamic and stage-specific regulation of astrocytic responses throughout the demyelination process (Fig. 7 Ci-ii).

Collectively, these results reveal distinct temporal gene expression patterns in the hippocampus during CPZ-induced demyelination, marked by early downregulation of OL and myelin genes and progressive upregulation of genes associated with astrogliosis, inflammation, and neurodegeneration.

### 3.7. Glutamatergic and GABAergic gene expression exhibits partial recovery between 6- and 12-Week CPZ treatment

mRNA transcript analysis revealed widespread downregulation of glutamatergic genes in the hippocampus following CPZ-induced demyelination (Fig. 7Di). Among the most downregulated across both timepoints were *Slc17a7* (VGLUT1; F(2,9) = 57.30; 6wk: *p* < 0.0001), *Gls* (F(2,9) = 6.844; 6wk: *p* = 0.0103), *Glul* (F(2,9) = 30.89; 6wk: p < 0.0001), *Gria4* (F(2,9) = 7.887; 6wk: *p* = 0.0073), *Shank2* (F(2,9) = 25.78; 6wk: *p* = 0.0001), and *Arl6ip5* (F(2,9) = 66.38; 6wk: p < 0.0001) *Grm1* (F (2,9) = 15.40; 6wk: *p* = 0.0007), *Grm2* (F(2,9) = 4.869; 6wk: *p* = 0.0261), *Grm7* (F(2,9) = 4.211; 6wk: *p* = 0.0467) were only downregulated at 6 weeks CPZ. These genes are involved in synaptic glutamate packaging (*Slc17a7*), enzymatic regulation of glutamate–glutamine cycling (*Gls*, *Glul)*, and post-synaptic scaffolding and receptor trafficking (*Shank2*, *Gria4*). *Grm1, 2 & 7* and *Arl6ip5* are associated with metabotropic receptor signaling and intracellular transport of glutamate transporters, respectively. Interestingly, *Slc1a3*, which encodes the glutamate transporter *EAAC1*, did not change (Fig. 7Di).

Comparative analysis between 6- and 12-week CPZ groups showed a significant increase in the expression of several glutamatergic genes at 12 weeks, including *Slc17a7* (*p* = 0.0019), *Gls* (p = 0.007), *Glul* (*p* = 0.0025), *Gria4* (*p* = 0.0137), *Grm1* (*p* = 0.0051), *Shank2* (*p* = 0.0045), and *Arl6ip5* (*p* = 0.0011), suggesting partial transcriptional recovery during chronic demyelination (Fig. 7Di).

Similarly, GABAergic gene expression was markedly downregulated at 6 weeks CPZ, preceding some recovery at 12 weeks (Fig. 7Dii). These included key regulators of GABAergic function such as *Kctd12* (F(2,9) = 17.11; 6wk: *p* = 0.0005), *Slc6a1* (GABA transporter; F(2,9) = 50.48; p < 0.0001), *Slc38a1* (glutamine transporter; F(2,9) = 29.55; p < 0.0001), *Gabra4* (GABAA receptor subunit; F(2,9) = 28.92; 6wk: p < 0.0001), *Gad1* (GABA synthesizing enzyme; F(2,9) = 13.45; *p* = 0.0012), *Gphn* (gephyrin; F(2,9) = 10.37; 6wk: *p* = 0.0028), *Cacna1b* (voltage-gated calcium channel; F(2,9) = 15.54; 6wk: p = 0.0007; 12wk: *p* = 0.0391), *Nsf* (F(2,9) = 36.45; 6wk: p < 0.0001), *Dnajc5* (F(2,9) = 17.03; 6wk: p = 0.0005), and *Rab3a* (F(2,9) = 19.66; 6wk: *p* = 0.0003) (Fig. 7Dii). These genes regulate presynaptic vesicle trafficking, calcium-dependent neurotransmission, and postsynaptic receptor stabilization.

Comparing 12wk to 6wk CPZ groups revealed increased expression of these GABAergic genes at the later timepoint: *Kctd12* (*p* = 0.0024), *Slc6a1* (*p* = 0.0013), *Slc38a1* (*p* = 0.0042), *Gabra4* (*p* = 0.0008), *Gad1* (*p* = 0.0089), *Gphn* (*p* = 0.0022), *Cacna1b* (*p* = 0.0063), *Nsf* (*p* = 0.0044), *Dnajc5* (*p* = 0.0079), and *Rab3a* (*p* = 0.0111) (Fig. 7Dii).

Together, these findings suggest that both glutamatergic and GABAergic systems undergo early transcriptional suppression during acute demyelination, with signs of delayed molecular recovery or compensation emerging by 12 weeks of CPZ treatment. This futile recovery phase may reflect adaptive changes in neurotransmitter systems in response to prolonged demyelination and seizure susceptibility.

## 4. Discussion

Seizures are caused by hypersynchronous neuronal firing, which generally stems from an imbalance between excitatory and inhibitory neurotransmission (Alldredge et al., 1989; Berkovic and Ottman, 2000; Singhi, 2011; Rana and Musto, 2018; Laing et al., 2022; Vezzani et al., 2022; Freiman et al., 2023; Sødal et al., 2024). While many factors can lead to seizures, including genetic, traumatic, metabolic, and inflammatory causes, about 2–3 % of individuals with MS also develop seizures (Koch et al., 2008; Calabrese et al., 2012; Nicholas et al., 2016; Mahamud et al., 2018; Mirmosayyeb et al., 2021; Grothe et al., 2023).

### 4.1. Seizures and MS: A closer look

In MS patients with seizures, the temporal lobe, particularly the hippocampus is often the most affected region (Calabrese et al., 2008; Ciolac et al., 2022). Cortical damage here, including demyelination and loss of inhibitory GABA interneurons, is strongly linked to seizure activity (Nicholas et al., 2016). Hippocampal demyelination is also associated with mesial temporal lobe epilepsy, where seizures often originate (Dutta et al., 2011; Grote et al., 2023). Postmortem studies show that, while overall neuronal loss in demyelinated hippocampi is minimal, synaptic density and the expression of proteins essential for axonal transport, synaptic plasticity, glutamate signaling, and memory are markedly reduced (Dutta et al., 2011). In RR-MS, patients with seizures exhibit more severe temporal lobe damage than those without, especially in the hippocampus, lateral temporal lobe, cingulate cortex, and insula (Calabrese et al., 2008).

PV+ interneurons, which are frequently myelinated (Micheva et al., 2016), are crucial for inhibitory control and synchronizing high-frequency network oscillations (Hasam-Henderson et al., 2018). Their function relies on intact myelination for rapid signal conduction (Stedehouder et al., 2018; Ramaglia et al., 2021; Dubey et al., 2022). Demyelination is associated with reduced numbers of this interneuron type in the hippocampal CA1 subfield (this study and (Lapato et al., 2017)) and likely their inhibitory capacity based on the loss of GAD67 expression observed in this study, ostensibly fostering epileptogenic conditions.

### 4.2. Glutamate dysregulation, demyelination, and seizures in MS

Seizures elevate extracellular glutamate, promoting excitotoxic injury. Chronic seizure activity drives adaptive changes in glutamate receptors and transporters, sustaining a pro-epileptogenic state (Barker-Haliski and White, 2015). Our findings show that MS patients with seizures (MS + S) exhibit hippocampal CA1 demyelination, astrogliosis, and reduced expression of the glutamate transporter GLT1 and water channel AQP4 (Lapato et al., 2020), changes previously linked to epilepsy (Proper et al., 2002; Binder et al., 2012; Lee et al., 2012; Hubbard et al., 2016; Peterson and Binder, 2019a, 2020; Peterson et al., 2021). Impaired astrocyte-mediated glutamate clearance likely contributes to excitotoxicity and seizure susceptibility.

Demyelination appears central to epileptogenesis in MS, creating a feedback loop in which seizures worsen neuroinflammation and further myelin loss. Yet, whether demyelination alone can trigger seizures remains unclear. The cuprizone (CPZ) model, which induces demyelination without adaptive immune responses, shows that chronic demyelination can produce seizures (Kesterson and Carlton, 1972), abnormal EEG activity, GABAergic interneuron loss, astrogliosis, reduced AQP4, and hippocampal neurodegeneration (Hoffmann et al., 2008; Lapato et al., 2017). While these findings strengthen the demyelination–seizure link, the timing and molecular triggers of seizure onset in demyelinated tissue remain unresolved.

This study examined how hippocampal demyelination drives seizure onset by integrating behavioral, EEG, molecular, and transcriptomic analyses across CPZ timepoints. We identified structural and molecular changes that precede seizures, independent of overt inflammation, highlighting potential mechanisms and therapeutic targets for MS-related epilepsy. Spontaneous seizures emerged after 6 weeks of CPZ exposure—a critical phase marked by partial remyelination in some brain regions but persistent hippocampal demyelination (Matsushima and Morell, 2001; Koutsoudaki et al., 2009). Seizure incidence correlated with demyelination severity, suggesting that incomplete myelin repair alongside ongoing damage fosters network hyperexcitability. We propose that while certain hippocampal circuits attempt repair, others become prone to seizure generation. Future studies will assess neuroprotective pathways to further clarify this transition.

### 4.3. Structural decline of CA1 and depletion of inhibitory neurons

CA1 pyramidal neurons are glutamatergic and integrate distinct inputs through their spatially segregated dendritic domains. The distal apical dendrites receive long-range excitatory input from the entorhinal cortex and thalamic nucleus reuniens, while the basal and proximal apical dendrites primarily receive input from CA3 neurons via the Schaffer collaterals. In mice, basal dendrites reside in the stratum oriens and apical dendrites in the SR (Benavides-Piccione et al., 2020). The CA1 region, highly vulnerable in both epilepsy (Meier et al., 1992; Lehmann et al., 2000; Cavazos et al., 2004; Yang et al., 2022) and MS (Sicotte et al., 2008; Papadopoulos et al., 2009; Ziehn et al., 2010; Nicholas et al., 2016; Planche et al., 2018; Lapato et al., 2020). During CPZ exposure, structural decline was seen by 12 weeks where the pyramidal layer was narrowed, dendritic complexity was reduced, and PV+ interneuron counts in the SP were significantly decreased in past and present study (Lapato et al., 2017). These changes suggest that demyelination disrupts excitatory–inhibitory balance through selective neuronal and glial alterations, increasing seizure susceptibility via excitotoxic mechanisms.

### 4.4. Altered glutamate homeostasis in demyelination and epileptogenesis

Glutamate, the brain’s main excitatory neurotransmitter, is essential for normal neuronal function (Dutta et al., 2011) but can cause excitotoxic injury when elevated (Proper et al., 2002; Sarac et al., 2009). In epilepsy, hippocampal glutamate surges often precede seizures (During and Spencer, 1993; Sarlo and Holton, 2021), though patterns vary with region and brain state (Soukupova et al., 2015; Buchanan et al., 2016; Sarlo and Holton, 2021). In MS, inflammatory activity can exacerbate glutamate release, contributing to lesion formation (Azevedo et al., 2014). While glutamate dysregulation is well recognized in other neurological disorders, its direct link to demyelination has been unclear.

These findings parallel prior reports of region-specific glutamate alterations in CPZ models and MS patients, though variability across brain regions and detection methods remains a challenge. Our bulk hippocampal assays provide only static snapshots, underscoring the need for real-time, region-specific monitoring. Real-time biosensor monitoring (Moriyama et al., 2025) will be critical to define how demyelination-driven increases in glutamate induce seizures. Our findings support a role for demyelination-induced glutamate dysregulation in driving hyperexcitability and seizure susceptibility. Prior CPZ studies report variable glutamate changes depending on brain region and detection method—ranging from transient whole-brain spikes (An et al., 2021) to persistent regional increases on GluCEST MRI (Lee et al., 2020). In MS, glutamate is often elevated in CSF and acute lesions (Stover et al., 1997; Sarchielli et al., 2003; Srinivasan et al., 2005; Klauser et al., 2018), but reduced in the hippocampus (Gao et al., 2018).

Our study shows that hippocampal demyelination alone disrupts glutamate homeostasis—affecting transporters, enzymes, and extracellular levels. Glutamate rose early (3 weeks CPZ), dipped slightly at 6 weeks during initial seizure onset, and increased sharply by 9–12 weeks, coinciding with high seizure incidence. Levels at 12 weeks (~2.2 μM) were sufficient to cause chronic excitotoxicity, suggesting an early window of vulnerability followed by failed compensation during chronic demyelination. Because our measurements capture only static snapshots from hippocampal homogenates, the true temporal and spatial dynamics remain unclear. Real-time, region-specific monitoring with glutamate biosensors will be essential to directly link demyelination-related glutamate elevations to seizure initiation and to reconcile differences between MS and CPZ models.

### 4.5. Altered expression of glutamate transporters and metabolic enzymes

Glutamate homeostasis is maintained through tightly coordinated clearance by neurons and astrocytes (Danbolt, 2001), with astrocytes removing ~80 % of extracellular glutamate via high-affinity transporters such as GLT1 (EAAT2) and GLAST. In the hippocampus, astrocytic coverage of glutamatergic synapses varies, and in the CA1 stratum radiatum (SR) only ~57 % of Schaffer collateral–CA1 synapses are ensheathed by astrocytes (Parpura and Verkhratsky, 2012). The water channel AQP4 supports glutamate-induced astrocytic swelling, linking osmotic regulation to excitatory neurotransmission (Szu and Binder, 2022). Once cleared, glutamate is converted to glutamine by GS in astrocytes, shuttled back to presynaptic neurons via sodium-coupled neutral amino acid transporters (SNAT), reconverted by glutaminase, and packaged into vesicles by VGLUTs. GAD67, largely neuronal, converts glutamate to GABA, maintaining inhibitory tone (reviewed in (Parpura and Verkhratsky, 2012)).

In MS, elevated CSF glutamate (Sarchielli et al., 2003) and reduced hippocampal EAAT1/EAAT2 have been reported, alongside increased VGLUT expression in lesions (Vercellino et al., 2007; Gautier et al., 2015). Similar patterns occur in epilepsy, where sclerotic hippocampi display EAAT2 loss (Proper et al., 2002). Our Western blot analysis of whole hippocampi from CPZ-treated mice (6–12 weeks) detected no changes in GLT1, GLAST, or VGLUT1, likely due to subregion-specific effects masked in bulk tissue. This limitation highlights the importance of spatially resolved methods. For instance, studies using synaptosomal and membrane fractionation have revealed significant GLT1 loss specifically at the tripartite synapse in the intrahippocampal kainic acid model of temporal lobe epilepsy (Peterson and Binder, 2019a).

We resolved subregional protein expression differences in CA1 SR & SP laminae using IHC. During demyelination, both layers showed robust astrogliosis, consistent with increasing evidence implicating reactive astrocytes in epileptogenesis (Vezzani et al., 2022). GLT1 expression was significantly reduced in SP and SR despite appearing redistributed to more astrocytes suggesting impaired transporter capacity per cell (reviewed in (Peterson and Binder, 2019b)). GLAST downregulation occurred later, first in SP (9–12 weeks) and then in SR (12 weeks). GS expression remained stable in SP but increased in SR at 9 weeks, contrasting with previous epilepsy-related GS loss (Eid et al., 2004; Eid et al., 2008; Sandhu et al., 2021). and indicating possible region-specific compensation. AQP4 was elevated in SP at 6 weeks but decreased in SR at 9–12 weeks, consistent with spatially divergent vulnerability (Binder et al., 2012). VGLUT1 remained unchanged in SP but showed modest SR reductions at 6–9 weeks, hinting at altered vesicular glutamate loading. GAD67 decreased markedly in both SP and SR during chronic demyelination, suggesting reduced inhibitory tone and a shift toward hyperexcitability. Together, these findings reveal layer-specific and temporally distinct changes in glutamate transporters, metabolic enzymes, and inhibitory markers that likely interact to promote excitotoxicity and epileptogenesis in the demyelinated hippocampus.

### 4.6. Longitudinal changes in hippocampal gene expression during demyelination

To complement the protein data, we conducted whole-hippocampal mRNA profiling. In the 6-week CPZ group, most OL and myelin-associated genes were significantly reduced, consistent with decreased MBP immunoreactivity in CA1. By 12 weeks, many of these genes recovered; however, *Mag, Mbp, Myrf, Pllp, Plp1, Sox10*, and *Ugt8a* remained low. Interestingly, Bcas1, Dlx2, and Olig2 were elevated at 12 weeks, suggesting expansion of progenitor populations and a possible endogenous remyelination attempt.

Neuronal structural and synaptic genes showed a similar biphasic pattern—broad downregulation at 6 weeks (except *Ngf*) followed by significant recovery at 12 weeks, with Ngf and Syngr1 maintaining stable or elevated expression.

Astrocytic alterations, supported by increased CA1 GFAP immunoreactivity, were reflected in transcript data: *Gfap* and proinflammatory markers (*Vim, C1qa, C4a/b, Serpina3n*) were upregulated at both 6 and 12 weeks. Serpina3n, known for its role in NF-κB–mediated inflammation (Liu et al., 2023). and previously elevated in kainic acid–induced epilepsy and chronic EAE, was similarly increased here. Other astrocytic genes (*AldH1a, AldH1/1, Amigo2*, and *Trim* family members) were suppressed at 6 weeks but recovered by 12 weeks. In contrast, neuroprotective astrocytic genes (*S100a10, Aqp4*) remained downregulated, indicating loss of supportive glial functions.

Glutamatergic and GABAergic transcripts were broadly reduced at 6 weeks—coinciding with seizure onset—followed by partial recovery at 12 weeks. *Slc17a7* (VGLUT1) and *Gad1* (GAD67) were decreased at both gene and protein levels, implicating impaired glutamate release and GABA synthesis. Discrepancies emerged for *Slc1a3* (GLAST), whose mRNA was unchanged despite protein loss during chronic demyelination. Additional dysregulated genes have established links to excitotoxicity and epilepsy: *Shank2*, a postsynaptic density scaffolding protein critical for excitatory synapse maintenance, whose mutations cause synaptic disruption and NMDAR signaling defects (Gong et al., 2009; Schmeisser et al., 2012; Won et al., 2012). *Grin2a* and *Grm7*, encoding glutamate receptor subunits, are associated with epilepsy, with *Grm7* mutations linked to hypomyelination and progressive demyelination (Addis et al., 2017; Strehlow et al., 2019). *Slc6a1*, encoding a GABA transporter, where loss-of-function reduces GABA reuptake and promotes epileptiform activity. *Gabra4*, encoding a GABA_A_ receptor subunit, associated with neonatal epilepsy and severe developmental delay (Carvill et al., 2015; Palmer et al., 2016). Feasibly, the transcriptional changes observed in this study could parallel the epileptogenic potential associated with these genes and future studies will tie mRNA changes to protein expression and localization.

Mice exposed to the CPZ diet exhibit a markedly higher incidence of seizures (up to 88 % at 12 weeks) compared to MS patients, in whom the reported incidence is approximately 3 % (Catenoix et al., 2025). This discrepancy likely reflects the widespread and reproducible demyelination induced by CPZ across both gray and white matter regions including the cortex, hippocampus, corpus callosum, and cerebellum—whereas demyelinating lesions in MS are heterogeneously distributed and seizure incidences underreported (Catenoix et al., 2025). This underreporting is potentially caused by the difficulty of distinguishing between epileptic seizures and other MS-related symptoms, as well as patient and clinician misinterpretation of seizure events.

Significant demyelination of brain structures is evident by 3 weeks of CPZ diet, and by 4–5 weeks, over 90 % of axons are demyelinated (Matsushima and Morell, 2001). Our results show a marked increase in hippocampal glutamate levels in 3 weeks of cuprizone diet. In the context of demyelination, excess glutamate may further exacerbate demyelination through multiple mechanisms: (i) excitotoxic injury and death of OLs and neurons leading to increased axonal demyelination; (ii) disruption of PV interneuron myelination and function, impairing inhibitory tone (Dubey et al., 2022); and (iii) activation of astrocytes and microglia, which release cytotoxic mediators and impair glutamate uptake (Gudi et al., 2014). Together, these processes likely create a self-reinforcing cycle of inflammation, excitotoxicity, and demyelination that contributes to persistent network hyperexcitability and increased seizure susceptibility.”

**Fig. 8.**
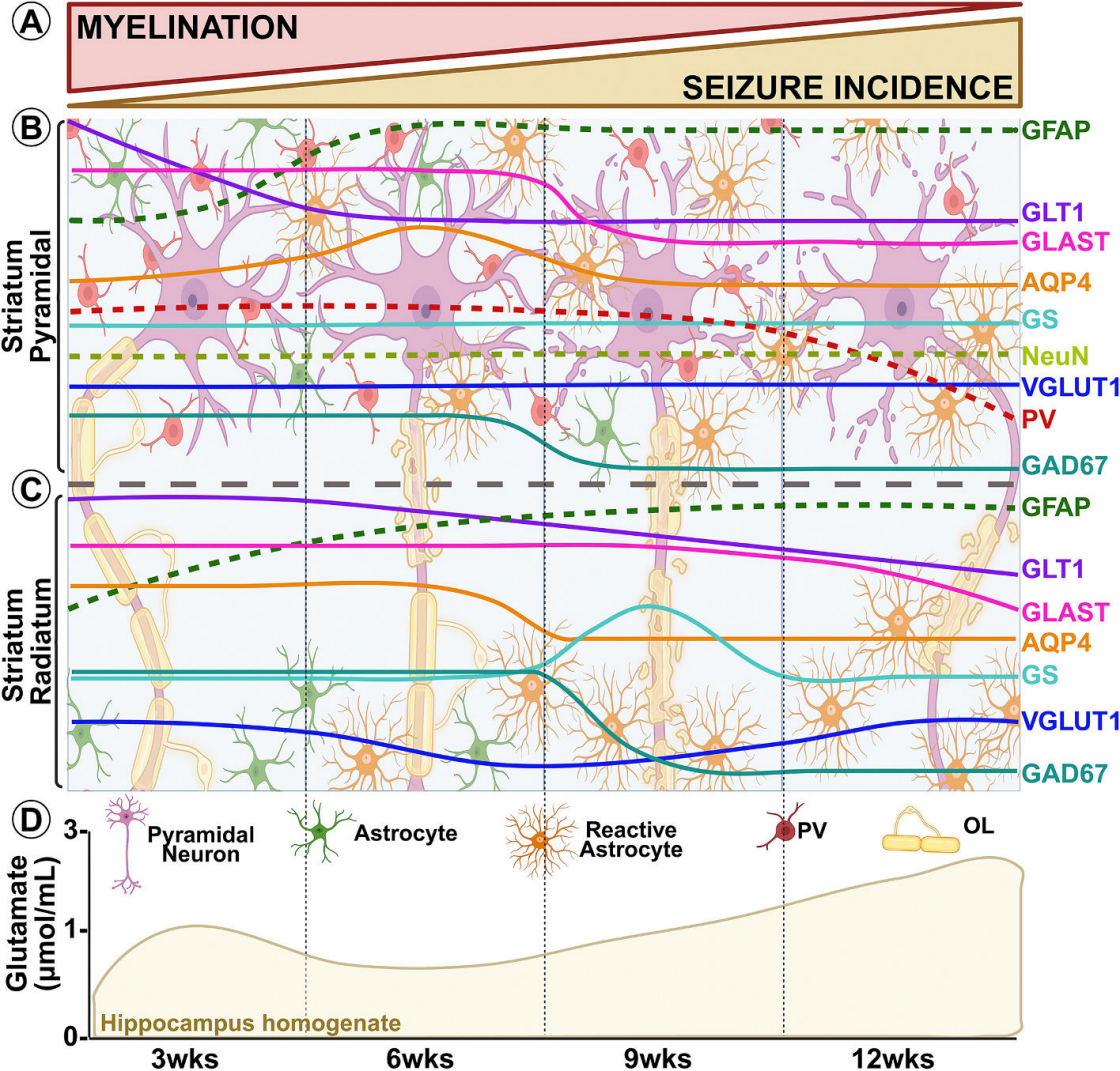
Model summarizing demyelination-induced glutamate dysregulation and increased incidence of spontaneous seizures. This model illustrates how chronic demyelination in the hippocampus, induced by CPZ treatment, leads to glutamate dysregulation and the development of spontaneous seizures. (A) Seizure Incidence and Demyelination: As demyelination progresses, we observe a corresponding increase in the incidence of spontaneous seizures. (B–C) Structural and Molecular Changes Leading to Excitatory/Inhibitory Imbalance: The hippocampus of demyelinated mice exhibits several structural and molecular alterations contributing to an excitation/inhibition imbalance at the synapse. These alterations include 1. CA1 pyramidal neuron layer atrophy and decreased dendritic arborization, 2. a loss in inhibitory PV+ interneurons, and 3. Sustained astrogliosis. Whole hippocampus western blot analysis showed no difference between control and demyelinating groups. However, IHC analysis of the CA1 SP and SR revealed specific layer and temporal changes. At the onset of seizures, astrogliosis developed and persisted concurrently with decreased expression of GLT1 and GLAST, suggesting disrupted glutamate uptake. Though AQP4 expression is increased in the SP at 6 weeks, it returns to normal and even drops in the SR during chronic demyelination, compounding the effect of hindered astrocyte-mediated glutamate uptake. To convert sequestered (collected) glutamate to glutamine, astrocytic GS increased in the SR at 9 weeks, but it did not sustain. No change in neuron density or VGLUT1 in the SP was apparent, but a slight decrease in the SR at 6 and 9 weeks may allude to redistribution of glutamate vesicular loading transporter along demyelinated axons. By chronic demyelination, PV+ interneurons and GAD67 are significantly reduced, further demonstrating the growing imbalance of excitation over inhibition. Transcriptomic analysis revealed major wide-spread downregulation of genes at 6 weeks. However, many neuronal, astrocytic, glutamatergic, and GABAergic genes attempt to recover normal expression, highlighting multiple compensatory mechanisms at work. (D) Insufficient Compensatory Mechanisms: Despite the adaptive responses at the protein and gene level, the redistribution of glutamate regulatory proteins is insufficient to restore homeostasis. The cumulative effect is persistent glutamate dysregulation and continued spontaneous seizure activity.

In summary, our results show that chronic demyelination heightens seizure susceptibility by disrupting hippocampal excitatory–inhibitory balance through two converging mechanisms. These changes are presented in a visual summary in Fig. 8. First, demyelination-induced neuronal injury, including reduced dendritic complexity, drives excessive glutamate release while impairing clearance, largely due to GLT1 and GLAST downregulation. Second, inhibitory control is weakened by reduced expression of critical regulators such as GAD67 and parvalbumin. Although compensatory changes in transporter and enzyme expression emerge over time, they fail to fully restore network stability, allowing hyperexcitability and spontaneous seizures to persist by 12 weeks. To our knowledge, this is the first longitudinal CPZ study to integrate behavioral, electrophysiological, transcriptomic, and proteomic analyses of glutamate and GABA pathway components. These findings establish a mechanistic framework linking demyelination to excitotoxicity and point toward new therapeutic targets, which can be further validated using conditional transgenic approaches.

Fig. 8 was created in BioRender. Anderson, A. (2025) https://BioRender.com/27mgyb4

## Supplementary Material

Suppl Figures

RNA Gene List

## Data Availability

NanoString raw data has been deposited in NCBI’s Gene Expression Omnibus (Edgar *et al*., 2002) and is accessible through GEO Series accession number GSE308963 (https://www.ncbi.nlm.nih.gov/geo/query/acc.cgi?acc=GSE308963) Raw and fold change gene data have been submitted as Supplementary Tables. All EEG files are available on request.

## Appendix A. Supplementary data

Supplementary data to this article can be found online at https://doi.org/10.1016/j.nbd.2025.107125.

## Abbreviations:

MS: multiple sclerosis
DM: demyelination
EEG: electroencephalogram
CPZ: cuprizone diet
IHC: immunohistochemistry
GFAP: glial fibrillary acidic protein
AQP4: aquaporin-4
GLT-1: glutamate transporter-1
GLAST: glutamate aspartate transporter
